# Shannon Entropy in Configuration Space for Ni-Like Isoelectronic Sequence

**DOI:** 10.3390/e22010033

**Published:** 2019-12-25

**Authors:** Jianjie Wan, Na Guo

**Affiliations:** College of Physics and Electronic Engineering, Northwest Normal University, Lanzhou 730070, China

**Keywords:** Shannon entropy, configuration space, multiconfiguration Dirac–Hartree–Fock method, relativistic configuration interaction

## Abstract

Discrete Shannon entropy was introduced in view of the mathematical properties of multiconfiguration methods and then used to interpret the information in atomic states expressed by the multiconfiguration Dirac–Hartree–Fock wavefunction for Ni-like isoelectronic sequence. Moreover, the relationship between the concepts, including sudden change of Shannon entropy, information exchange, eigenlevel anticrossing, and strong configuration interaction, was clarified by induction on the basis of the present calculation of the energy structure for Ni-like isoelectronic sequence. It was found that there is an interesting connection between the change of Shannon entropies and eigenlevel anticrossings, along with the nuclear charge Z, which is helpful to conveniently locate the position of eigenlevel anticrossings and information exchanging and understand them from the point of view of information, besides the traditional physical concepts. Especially, it is concluded that in a given configuration space eigenlevel anticrossing is a sufficient and necessary condition for the sudden change of Shannon entropy, which is also a sufficient condition for information exchange.

## 1. Introduction

Information concepts have been used in the analysis of a large variety of physical concepts for a long time. As one of them, Shannon information entropy [1] has been employed to elucidate physical and chemical properties of nuclear, atomic, and molecular systems from an information theoretical point of view. Today, Shannon information plays a more and more important role in studying atomic and molecular properties.

In much of the literature on atomic and molecular physics, the Shannon information entropy was almost calculated from the one-electron wavefunction, which can elucidate the uncertainty of localization of electron cloud in position and momentum spaces. González-Férez and Dehesa [2] calculated the Shannon entropy according to the nonrelativistic one-electron wavefunction in the presence of the uniform magnetic and electric fields in order to indicate or predict the avoided-crossing phenomena of some excited states of hydrogen atom in the presence of magnetic and electric fields. In their work, a sudden change at the avoided-crossing region and an informational exchange between the states had been found. He et al. [3] also proposed a method to calculate the positions of avoided crossings for Rydberg potassium in a static electric field on the basis of Shannon entropy. Their work shows that the Shannon entropy is an efficient parameter for characterization and prediction of avoided crossings of Rydberg potassium. As far as we know, in atomic and nuclear physics, the information entropy has already been used to study the quantum chaotic system [4,5] by using configuration interaction method to analyze the spectrum and the eigenstates of complex atom and heavy nuclei, in which the wavefunction of the excited states are chaotic superpositions of hundreds or thousands of principal basis states.

On the other hand, as is well known, a number of level crossings have been found in the calculation of energy levels, along with various isoelectronic sequences. In early work, Fischer [6] described the level crossings and the oscillator-strength trends around the region of level crossings in detail. As mentioned in her work, the two energy curves for the upper and lower states are continuous and actually anticross. However, in atomic physics, it is customary to identify the level by the dominant component in an atomic state function like everywhere else. Traditionally speaking, in the study on the isoelectronic sequence, the level crossing, in fact, means state crossing which is denoted by the configuration state function that has the largest weight in the atomic state function. Therefore, whether it is a level crossing in an isoelectronic sequence or anticrossing (or avoided crossing) in an external field, the eigenlevel anticrossing would be used in order to avoid confusion in the present work.

To our knowledge and mentioned above, Shannon entropy has been used to analyze the quantum chaotic system (e.g., [4,5]) and indicate the extent of localization [2,3,7,8,9,10,11,12,13,14,15,16,17,18,19] and complexity [20,21,22,23,24,25] of an electronic cloud on the basis of the one-electron orbitals or density distribution. However, there is almost no discussion about the information in multiconfiguration methods, such as the multiconfiguration self-consistent field (MCSCF) and configuration interaction (CI) methods. In this paper, discrete Shannon entropy was introduced in order to measure information on the atomic states in the configuration space. In Section 2, we provide a complete description of the theoretical method. In Section 3, the Shannon entropies are shown for the ground and single excited states, along with the Ni-like isoelectronic sequence. Then, the relationship between the sudden change of Shannon entropy, information exchange, eigenlevel anticrossing, and strong configuration interaction is discussed based on the calculated energy levels, configuration mixing coefficients, and Shannon entropies. Finally, some concluding remarks and outlook are summarized in Section 4.

## 2. Theoretical Considerations

In the calculation on atomic structure, the wavefunction of atoms and ions can be obtained by the multiconfiguration method. Generally speaking, there are two major categories, i.e., multiconfiguration self-consistent field and configuration interaction methods according to whether the one-electron orbitals change in the calculation processes. On the other hand, there are also two main treatments, that is, in nonrelativistic and relativistic ways. In the nonrelativistic treatment, the configuration interaction with nonrelativistic orbital basis and multiconfiguration Hartree–Fock (MCHF) method with the one-electron orbitals generated by the self-consistent field procedure [26] have been employed. Likely, the relativistic configuration interaction (RCI) with relativistic one-electron orbitals and multiconfiguration Dirac–Hartree–Fock (MCDHF) methods with the relativistic electron orbitals generated by the self-consistent field procedure [27] have also been used to interpret the atomic states. Nonetheless, all of them have the same form:

(1) $$\begin{array}{c} |\Psi_{i}\left(J^{P}\right)\rangle =\sum_{j=1}^{n_{c}}C_{ij}|\Gamma_{j}\left(J^{P}\right)\rangle ,i=1,2,\cdots ,n_{c}, \end{array}$$

where $|\Psi_{i}\left(J^{P}\right)\rangle$ is the *i*-th atomic state function (ASF) corresponding to the *i*-th level. $|\Gamma_{j}\left(J^{P}\right)\rangle$, $j\;=\;1,2,\cdots ,n_{c}$ are the so-called configuration state functions (CSF), which can expand a subspace with the certain total angular momentum (J) and parity (P) in the whole configuration space (or infinite eigenstate space). In this paper, the even and odd parities are described by the superscripts *e* and *o*. $n_{c}$ is the number of the configuration state functions. In multiconfiguration methods, the atomic state functions are expressed as a linear combination of configuration state functions with the same symmetry and $C_{ij},j=1,2,\cdots ,n_{c}$ are the so-called configuration mixing coefficients for the *i*-th atomic state function, the modulus square of which can be usually used to indicate the weight of the *j*-th configuration in the *i*-th atomic state, yielding the normalization condition for the *i*-th atomic state function:

(2) $$\begin{array}{c} \sum_{j=1}^{n_{c}}{\left|C_{ij}\right|}^{2}=1,i=1,2,\cdots ,n_{c}. \end{array}$$

Traditionally, the atomic state or energy level is named after the dominant component in expansion of atomic state function, i.e., the configuration with the largest modulus square.

In our previous work [28], a discrete Shannon entropy was set up to measure the information in configuration space because of the properties of configuration mixing coefficients, $0\leq |C_{ij}{|}^{2}\leq 1,i$, $j\;=\;1,2,\cdots ,n_{c}$, that is, if we define the configuration weights

(3) $$\begin{array}{c} \rho_{ij}\equiv {\left|C_{ij}\right|}^{2}\in \left[0,1\right],i,j=1,2,\cdots ,n_{c}, \end{array}$$

then $s_{ij}=-\rho_{ij}$ln$\rho_{ij}$ is the information entropy for the *j*-th configuration state function $|\Gamma_{j}\left(J^{P}\right)\rangle$ in the *i*-th atomic state function $|\Psi_{i}\left(J^{P}\right)\rangle$ corresponding to a certain energy level, and it yields the normalization condition

(4) $$\begin{array}{c} \sum_{j=1}^{n_{c}}\rho_{ij}=1,i=1,2,\cdots ,n_{c}. \end{array}$$

Therefore, the Shannon entropy of the *i*-th energy level described by the $|\Psi_{i}\left(J^{P}\right)\rangle$ is defined by

(5) $$\begin{array}{c} S_{\Psi_{i}}=-\sum_{j=1}^{n_{c}}\rho_{ij}\ln\rho_{ij},i=1,2,\cdots ,n_{c}, \end{array}$$

which can be used to indicate the information on a certain energy level in quantity, i.e., the Shannon entropy can measure the uncertainty of the configurations for each certain atomic state in a given configuration space. According to its mathematical properties, it is easy to show that the range of the multiconfiguration wavefunction for the *i*-th atomic state is given by $0\leq S_{\Psi_{i}}\leq$ln$n_{c}$. The zero value is for the *i*-th atomic state, where one of the $\rho_{ij}$ equals 1 and all other $\rho_{ij}$ are zero, which supplies the most information in the configuration space from the point of view of information because of its least entropy. The maximum value results for uniformly weights of configuration states in the *i*-th atomic state function

(6) $$\begin{array}{c} \rho_{ij}=\frac{1}{n_{c}},j=1,2,\cdots ,n_{c}. \end{array}$$

The above situation is a limit of the strongest configuration interaction, which implies there is no dominant configuration state wavefunction in the configuration space. In a word, in this situation the weights are distributed equally into each CSF in the subspace; therefore, the atomic state wavefunction has the most uncertain information.

In the present work, the energy levels of ground and single excited states of Ni-like isoelectronic sequence were calculated by using the multiconfiguration Dirac–Hartree–Fock and relativistic configuration interaction methods [27]. As is well known, Ni-like ions have 28 electrons that constitute their ground configuration of 1s${}^{2}$2s${}^{2}$2p${}^{6}$3s${}^{2}$3p${}^{6}$3d${}^{10}$${}^{1}$S${}_{0}$. In order to calculate the energy levels, we constructed the subspaces for calculating the energy levels of Ni-like ions, which have been shown in detail in Table 1 and Table 2, where the closed subshells were conveniently omitted. For simplicity, only the ground and single excited configurations were included in this calculation, where there are 51 and 56 configuration state functions with even and odd parity, respectively. Within the framework of multiconfiguration Dirac–Hartree–Fock method, the radial orbitals were firstly generated from Dirac–Hartree–Fock equations by a self-consistent field procedure. Next, the relativistic configuration interaction calculation was carried out to obtain the resultant energies, which include Breit interaction, finite nucleus and the lowest-order QED correction, and corresponding mixing coefficients in the configuration space which can be expanded by the configuration basis set that were made of the relativistic electronic orbitals mentioned above. By the way, the calculated values of energy levels will not be given again in this paper, which had been compared with other theoretical and experimental results within the relative accuracy of about 1% or less [29,30,31], especially for highly charged ions. Finally, the Shannon entropy for each energy level can be easily obtained from the configuration mixing coefficients according to Equations (3) and (5).

## 3. Results and Discussion

**Figure 1:**
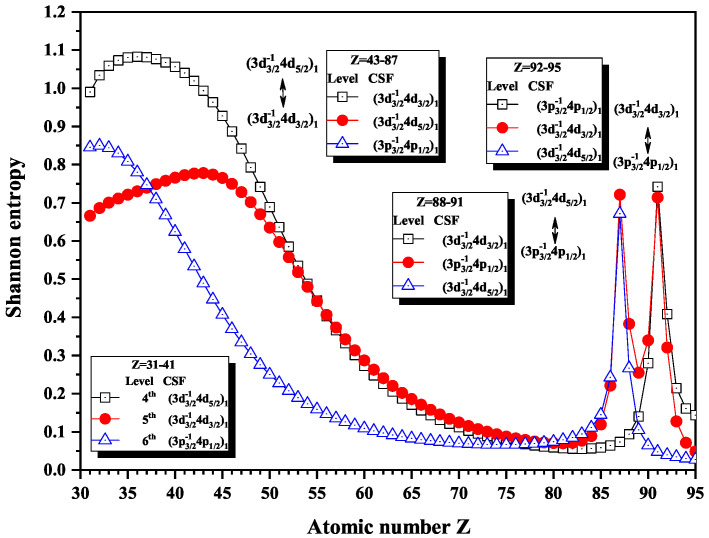
Shannon entropies for the 4th, 5th, and 6th levels labeled as (3d${}_{3/2}^{-1}$4d${}_{5/2}{)}_{1}$, (3d${}_{3/2}^{-1}$4d${}_{3/2}{)}_{1}$, and (3p${}_{3/2}^{-1}$4p${}_{1/2}{)}_{1}$ in the subspace with $J^{P}=1^{e}$ for Ni-like isoelectronic sequence with Z = 31–95.

**Figure 2:**
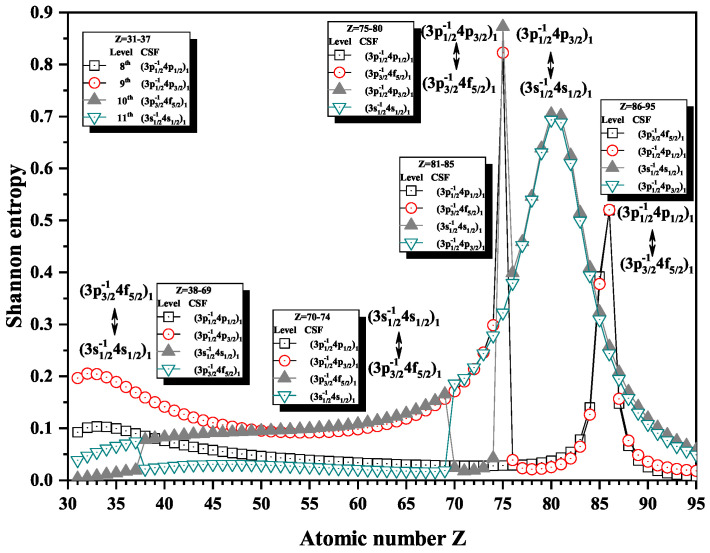
Shannon entropies for the 8th, 9th, 10th, and 11th levels labeled as (3p${}_{1/2}^{-1}$4p${}_{1/2}{)}_{1}$, (3p${}_{1/2}^{-1}$4p${}_{3/2}{)}_{1}$, (3p${}_{3/2}^{-1}$4f${}_{5/2}{)}_{1}$, and (3s${}_{1/2}^{-1}$4s${}_{1/2}{)}_{1}$ in the subspace with $J^{P}=1^{e}$ for Ni-like isoelectronic sequence with Z = 31–95.

Over the past three decades, systematic trends have been found in energy levels, oscillation strengths, radiative and nonradiative probabilities and lifetimes, and so on along an isoelectronic sequence, which have the same number of electrons in atomic systems with different nuclear charge Z. In the present work, we calculated the Shannon entropies of the ground and singly excited states for Ni-like isoelectronic sequence and focused on the relationship between the sudden change of Shannon entropy, eigenlevel anticrossing, information exchange, and strong configuration interaction. Figures 1, 2, 5–9 and 11–18 present the Shannon entropies for the levels in the corresponding configuration space, and the dominant configuration of each level is given in these figures according to the configuration mixing coefficients. In these figures, the marks are arranged from up to down in order to describe the eigenlevels of different ions in the corresponding subspace in Ni-like isoelectronic sequence.

Figure 1 and Figure 2 show the Shannon entropies and the dominant components for seven $J^{P}=1^{e}$ levels of Ni-like isoelectronic sequence; some of these were discussed in the preliminary work [28], and a rough conclusion was drawn. The present work shows more detailed analysis and corrected results. In Figure 1, the Shannon entropies have maxima at Z =87 and 91 for the 6th and 4th levels, respectively, while the 5th level has two maxima at Z =87 and 91. The peaks are considered as the first kind of sudden change of Shannon entropies in the present work. Apparently, the 4th level has one peak, the 5th two, and the 6th one. From the perspective of information, the information of eigenlevels is exchanged near the mentioned peaks position. Definitely, the 4th, 5th, and 6th levels are, in turn, labeled by their dominant components (3d${}_{3/2}^{-1}$4d${}_{3/2}{)}_{1}$, (3d${}_{3/2}^{-1}$4d${}_{5/2}{)}_{1}$, and (3p${}_{3/2}^{-1}$4p${}_{1/2}{)}_{1}$ in configuration subspace with $J^{P}=1^{e}$ for nuclear charge Z =43 to 87. At Z =88, the dominant components (3d${}_{3/2}^{-1}$4d${}_{5/2}{)}_{1}$ and (3p${}_{3/2}^{-1}$4p${}_{1/2}{)}_{1}$ exchanged, and these levels are labeled as (3d${}_{3/2}^{-1}$4d${}_{3/2}{)}_{1}$, (3p${}_{3/2}^{-1}$4p${}_{1/2}{)}_{1}$, and (3d${}_{3/2}^{-1}$4d${}_{5/2}{)}_{1}$ from Z =88 to 91. Further, the dominant components (3d${}_{3/2}^{-1}$4d${}_{3/2}{)}_{1}$ and (3p${}_{3/2}^{-1}$4p${}_{1/2}{)}_{1}$ swap with each other at Z = 92, so the levels are named (3p${}_{3/2}^{-1}$4p${}_{1/2}{)}_{1}$, (3d${}_{3/2}^{-1}$4d${}_{3/2}{)}_{1}$, and (3d${}_{3/2}^{-1}$4d${}_{5/2}{)}_{1}$. In other words, the information of the 5th level exchanges, in turn, with the 6th and 4th levels at Z =88 and 92.

**Figure 3:**
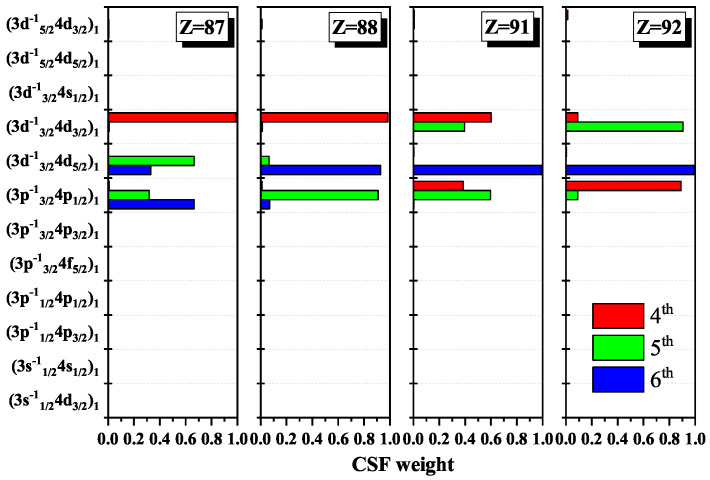
Weights of configuration state function in the configuration space with $J^{P}=1^{e}$ for the 4th, 5th, and 6th levels of Ni-like ions with Z =87, 88, 91, and 92.

In order to show the information exchange between the levels at some certain Z, Table 3 and Figure 3, respectively, present the configuration mixing coefficients and weights of configuration state functions for the atomic state functions in the subspace with $J^{P}=1^{e}$ corresponding to the 4th, 5th, and 6th levels in Figure 1. The weights of the dominant components (3d${}_{3/2}^{-1}$4d${}_{3/2}{)}_{1}$ for the 4th level, (3d${}_{3/2}^{-1}$4d${}_{5/2}{)}_{1}$ for the 5th level, and (3p${}_{3/2}^{-1}$4p${}_{1/2}{)}_{1}$ for the 6th level of Fr${}^{59+}$ ion (Z =87) are 98.9%, 66.6%, and 66.5%, respectively. It is clear that the information of the 5th and 6th levels in configuration space has not been concentrated one configuration, approximately, on the contrary of the 4th level, so the dominant components in the 5th and 6th levels have not been clear at Z =87. Then, the dominant components swap with each other at Z =88, so the information exchanged between the 5th and 6th levels around the positions with the maxima of Shannon entropies. It can be induced that, where there is a sharp maximum in Shannon entropy, there is an information exchange.

**Figure 4:**
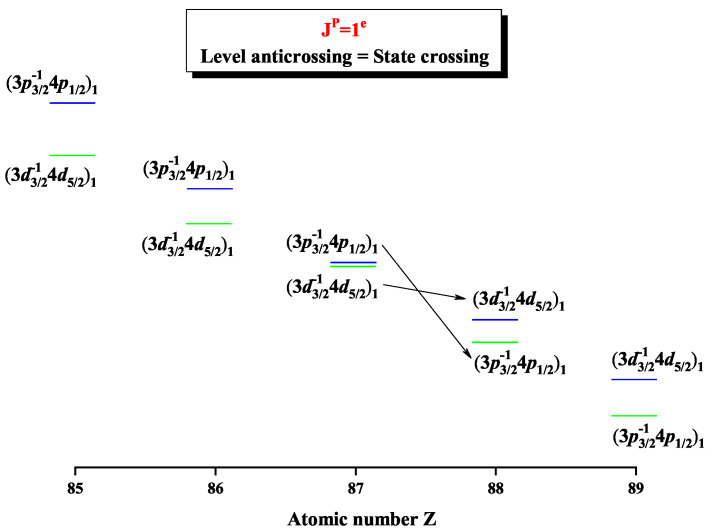
Energy diagrams for the 5th and 6th levels labeled as (3d${}_{3/2}^{-1}$4d${}_{5/2}{)}_{1}$ and (3p${}_{3/2}^{-1}$4p${}_{1/2}{)}_{1}$ in the subspace with $J^{P}=1^{e}$ for Ni-like isoelectronic sequence with Z = 85–89.

On the other hand, besides the redistribution of the configuration mixing coefficients and weights in configuration space, along with the nuclear charge Z, these peaks are also the reflection of eigenlevel anticrossings at Z =87 and 91, where the energy differences reach the minima, i.e., $\Delta E_{6,5}=0.2609$ Hartree and $\Delta E_{5,4}=0.3299$ Hartree, which are 5 orders of magnitude smaller than their energy eigenvalues. In Figure 4, as a schematic diagram, the difference between the 6th and 5th levels reaches a minimum at Z =87. Obviously, it can be found that the region of anticrossings can be easily and directly determined with the help of analysis on the Shannon entropies, along with the isoelectronic sequence of Ni-like ions. Furthermore, the configuration interaction is the strongest between (3d${}_{3/2}^{-1}$4d${}_{5/2}{)}_{1}$ and (3p${}_{3/2}^{-1}$4p${}_{1/2}{)}_{1}$ in Fr${}^{59+}$ ion (Z =87) and between (3d${}_{3/2}^{-1}$4d${}_{3/2}{)}_{1}$ and (3p${}_{3/2}^{-1}$4p${}_{1/2}{)}_{1}$ in Pa${}^{63+}$ ion (Z = 91). That is, strong configuration interaction can make information obscure and further may lead to information exchange in the configuration space so that the corresponding Shannon entropies reach the maxima for the 5th and 6th levels at Z =87 and for the 4th and 5th levels at Z = 91.

Exceptionally, there is no obvious changes in the entropies for the 4th and 5th levels around Z = 43. And it can also be found that the dominant components (3d${}_{3/2}^{-1}$4d${}_{5/2}{)}_{1}$ and (3d${}_{3/2}^{-1}$4d${}_{3/2}{)}_{1}$ exchange for the 4th and 5th levels at Z = 43, and both of the 4th and 5th levels have the same dominant component (3d${}_{3/2}^{-1}$4d${}_{3/2}{)}_{1}$ at Z = 42 from Table 3. Previously, we believed that strong configuration interaction can result in the anticrossing. However, the anticrossing does not take place between the two levels. It is obvious that the information exchange is not necessarily caused by strong configuration interaction that also need not lead to the eigenlevel anticrossing.

In our previous work [28], we also discussed the Shannon entropies for the levels labeled as (3p${}_{1/2}^{-1}$4p${}_{1/2}{)}_{1}$, (3p${}_{1/2}^{-1}$4p${}_{3/2}{)}_{1}$, (3p${}_{3/2}^{-1}$4f${}_{5/2}{)}_{1}$, and (3s${}_{1/2}^{-1}$4s${}_{1/2}{)}_{1}$ ranging from Z = 68 to 95 and roughly found the connection between the sudden changes of Shannon entropy and anticrossings. Here, we expand the region from Z = 31 to 95 in order to show complete information exchanges and anticrossings for the levels with $J^{P}=1^{e}$. Figure 2 gives the Shannon entropies of other four levels labeled as (3p${}_{1/2}^{-1}$4p${}_{1/2}{)}_{1}$, (3p${}_{1/2}^{-1}$4p${}_{3/2}{)}_{1}$, (3p${}_{3/2}^{-1}$4f${}_{5/2}{)}_{1}$, and (3s${}_{1/2}^{-1}$4s${}_{1/2}{)}_{1}$, where the exchanges of dominant component take place at Z = 38, 70, 75, 81, and 86, in turn, according to the position of sudden changes. That is, the exchanges of dominant component take place at Z = 38, 70, 75, 81, and 86, which can easily be seen from Table 4 and Table 5. Meanwhile, there are five anticrossings around those ions with Z = 38, 70, 75, 81, and 86. The minima of the energy differences are, respectively, $\Delta E_{11,10}=0.00615$ Hartree at Z = 37, $\Delta E_{11,10}=0.0792$ Hartree at Z = 70, $\Delta E_{10,9}=0.0613$ Hartree at Z = 75, $\Delta E_{11,10}=0.9544$ Hartree at Z = 80, and $\Delta E_{9,8}=0.3714$ Hartree at Z = 86. It is interesting that the kinds of changes for Shannon entropy in Figure 2 are richer than that in Figure 1 mentioned in our previous work [28]. Besides the narrow peak, there are simple jump-style changes at Z = 38 and 70 and overlapping narrow peak around Z = 80. Comparing with the previous results [28], more complete information is given in this paper, where some results have also been corrected.

At Z = 38 and 70, where the continuity of entropies is not maintained for the two levels, the Shannon entropies jump either up or down. This step is related to the information exchange for the corresponding levels more directly than that in the situation of maxima. It is found from Table 4 that the weights of the dominant components (3p${}_{3/2}^{-1}$4f${}_{5/2}{)}_{1}$ for the 10th level and (3s${}_{1/2}^{-1}$4s${}_{1/2}{)}_{1}$ for the 11th level in Figure 2 for Rb${}^{9+}$ ion (Z = 37) are 99.7% and 98.9%, while the weights of the dominant components (3s${}_{1/2}^{-1}$4s${}_{1/2}{)}_{1}$ for the 10th level and (3p${}_{3/2}^{-1}$4f${}_{5/2}{)}_{1}$ for the 11th level for Sr${}^{10+}$ ion (Z = 38) are 98.8% and 99.7%. Similarly, the weights of the dominant components (3s${}_{1/2}^{-1}$4s${}_{1/2}{)}_{1}$ for the 10th level and (3p${}_{3/2}^{-1}$4f${}_{5/2}{)}_{1}$ for the 11th level in Figure 2 of Tm${}^{41+}$ ion (Z = 69) are 96.6% and 99.8%, while the weights of the dominant components (3p${}_{3/2}^{-1}$4f${}_{5/2}{)}_{1}$ for the 10th level and (3s${}_{1/2}^{-1}$4s${}_{1/2}{)}_{1}$ for the 11th level of Yb${}^{42+}$ ion (Z = 70) are 99.7% and 96.0%. The common property is that all of the weights are approximate to one so that the Shannon entropies would be almost equal to zero for those levels, which shows the unambiguous level information from the configuration space and that the simple jump-style changes are not related to the strong configuration interaction. Furthermore, there is no direct relationship between the sudden changes of Shannon entropy and configuration interaction. But, this result obviously cannot be understood in terms of strong configuration interaction, so it is impled that this type of information exchanges may be caused by radial orbital mutations between Z = 37 ∼ 38 and Z = 69 ∼ 70. Because the orbital basis, which are generated by multiconfiguration self-consistent field method, is different for different ions, obviously, information exchange and strong configuration interaction can be easily analyzed by using mixing coefficient lists and configuration weight graphs (see Table 3, Table 4 and Table 5 and Figure 3). So, in order to avoid repetition, only the description of Shannon entropies and configuration mixing coefficients are given below so as to find their corresponding relationship in the other configuration space.

**Figure 5:**
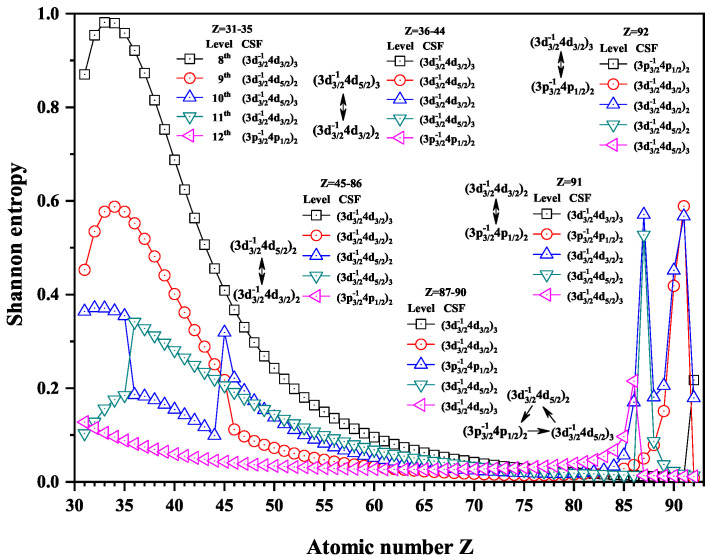
Shannon entropies for the 8th, 9th, 10th, 11th, and 12th levels labeled as (3d${}_{3/2}^{-1}$4d${}_{3/2}{)}_{3}$, (3d${}_{3/2}^{-1}$4d${}_{5/2}{)}_{2}$, (3d${}_{3/2}^{-1}$4d${}_{5/2}{)}_{3}$, (3d${}_{3/2}^{-1}$4d${}_{3/2}{)}_{2}$, and (3p${}_{3/2}^{-1}$4p${}_{1/2}{)}_{2}$ in the mixed subspace with $J^{P}=2^{e}$ and 3*^e^* for Ni-like isoelectronic sequence with Z = 31–92.

**Figure 6:**
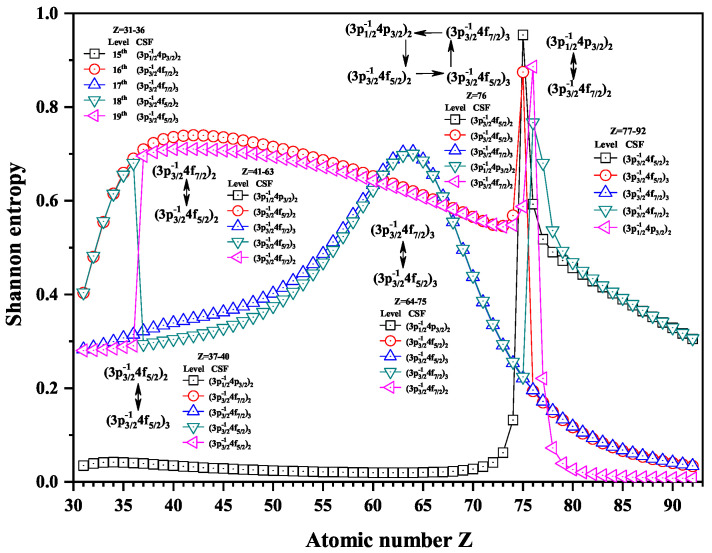
Shannon entropies for the 15th, 16th, 17th, 18th, and 19th levels labeled as (3p${}_{1/2}^{-1}$4p${}_{3/2}{)}_{2}$, (3p${}_{3/2}^{-1}$4f${}_{7/2}{)}_{2}$, (3p${}_{3/2}^{-1}$4f${}_{7/2}{)}_{3}$, (3p${}_{3/2}^{-1}$4f${}_{5/2}{)}_{2}$, and (3p${}_{3/2}^{-1}$4f${}_{5/2}{)}_{3}$ in the mixed subspace with $J^{P}=2^{e}$ and 3*^e^* for Ni-like isoelectronic sequence with Z = 31–92.

**Figure 7:**
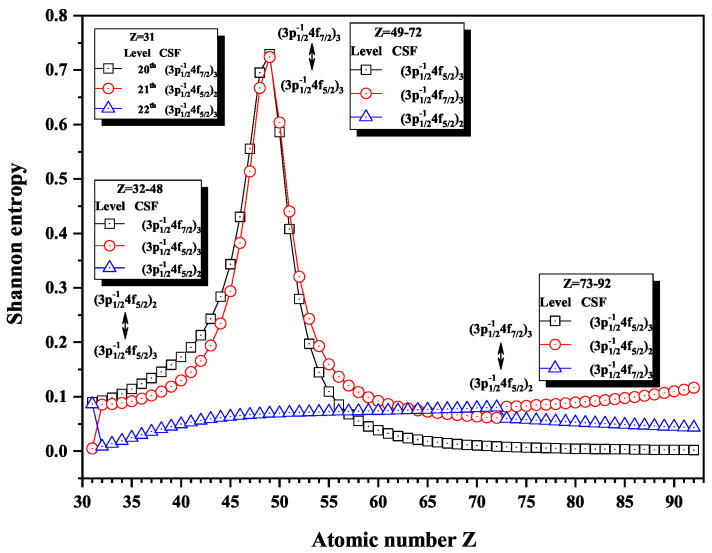
Shannon entropies for the 20th, 21st, and 22nd levels labeled as (3p${}_{1/2}^{-1}$4f${}_{7/2}{)}_{3}$, (3p${}_{1/2}^{-1}$4f${}_{5/2}{)}_{2}$, and (3p${}_{1/2}^{-1}$4f${}_{5/2}{)}_{3}$ in the mixed subspace with $J^{P}=2^{e}$ and 3*^e^* for Ni-like isoelectronic sequence with Z = 31–92.

Figure 5, Figure 6 and Figure 7 show the Shannon entropies for thirteen levels in a combined configuration space with $J^{P}=2^{e}$ and 3*^e^*. Figure 5 gives the Shannon entropies for the 8th, 9th, 10th, 11th, and 12th levels in the subspace. At Z = 36, the entropies jump down and up for the 10th and 11th levels, where the dominant components (3d${}_{3/2}^{-1}$4d${}_{5/2}{)}_{3}$ and (3d${}_{3/2}^{-1}$4d${}_{3/2}{)}_{2}$ exchange. At Z = 45, the entropies have a maximum for the 10th level besides jumping for the 9th and 10th, where the dominant components become (3d${}_{3/2}^{-1}$4d${}_{3/2}{)}_{2}$ and (3d${}_{3/2}^{-1}$4d${}_{5/2}{)}_{2}$. There is one maximum for the 12th level at Z = 86, one maximum for the 11th level at Z =87, two maxima for the 10th level at Z =87 and 90, and one maximum for the 9th level at Z = 90. According the sudden changes of entropies, it can also be found that the dominant components (3p${}_{3/2}^{-1}$4p${}_{1/2}{)}_{2}$, (3d${}_{3/2}^{-1}$4d${}_{5/2}{)}_{2}$, and (3d${}_{3/2}^{-1}$4d${}_{5/2}{)}_{3}$ have a triangle rotation for the 10th, 11th, and 12th levels at Z =87 for the first time, and then the 10th level exchanges its dominant components twice with the 11th and 9th levels, in turn, i.e., (3p${}_{3/2}^{-1}$4p${}_{1/2}{)}_{2}$ and (3d${}_{3/2}^{-1}$4d${}_{5/2}{)}_{2}$ at Z = 91 and (3d${}_{3/2}^{-1}$4d${}_{3/2}{)}_{2}$ and (3d${}_{3/2}^{-1}$4d${}_{5/2}{)}_{2}$ at Z = 92.

Figure 6 gives the Shannon entropies for the 15th, 16th, 17th, 18th, and 19th levels. The entropies jump at Z = 37 for the 18th and 19th levels, where the dominant components (3p${}_{3/2}^{-1}$4f${}_{5/2}{)}_{3}$ and (3p${}_{3/2}^{-1}$4f${}_{5/2}{)}_{2}$ exchange. However, there is an exception that the dominant components (3p${}_{3/2}^{-1}$4f${}_{5/2}{)}_{2}$ and (3p${}_{3/2}^{-1}$4f${}_{5/2}{)}_{2}$ exchange at Z = 41. There is an overlapping broad peak at Z = 64, where the dominant components (3p${}_{3/2}^{-1}$4f${}_{5/2}{)}_{3}$ and (3p${}_{3/2}^{-1}$4f${}_{7/2}{)}_{3}$ exchange, but the broad peak is not considered as a sudden change in Figure 6. At Z = 76, a quadrilateral rotation appears for the dominant components (3p${}_{1/2}^{-1}$4p${}_{3/2}{)}_{2}$, (3p${}_{3/2}^{-1}$4f${}_{5/2}{)}_{2}$, (3p${}_{3/2}^{-1}$4f${}_{5/2}{)}_{3}$ and (3p${}_{3/2}^{-1}$4f${}_{7/2}{)}_{3}$. At Z = 77, the dominant components (3p${}_{3/2}^{-1}$4f${}_{7/2}{)}_{2}$ and (3p${}_{1/2}^{-1}$4p${}_{3/2}{)}_{2}$ exchange.

In Figure 7, the entropies jump at Z = 32 and 73, and the 20th and 21st levels have maxima. At Z = 32, the dominant components (3p${}_{3/2}^{-1}$4f${}_{5/2}{)}_{3}$ and (3p${}_{3/2}^{-1}$4f${}_{5/2}{)}_{2}$ exchange with each other. At Z = 49, the dominant components (3p${}_{3/2}^{-1}$4f${}_{5/2}{)}_{3}$ and (3p${}_{3/2}^{-1}$4f${}_{7/2}{)}_{3}$ exchange. At Z = 73, (3p${}_{3/2}^{-1}$4f${}_{5/2}{)}_{2}$ and (3p${}_{3/2}^{-1}$4f${}_{7/2}{)}_{3}$ exchange.

**Figure 8:**
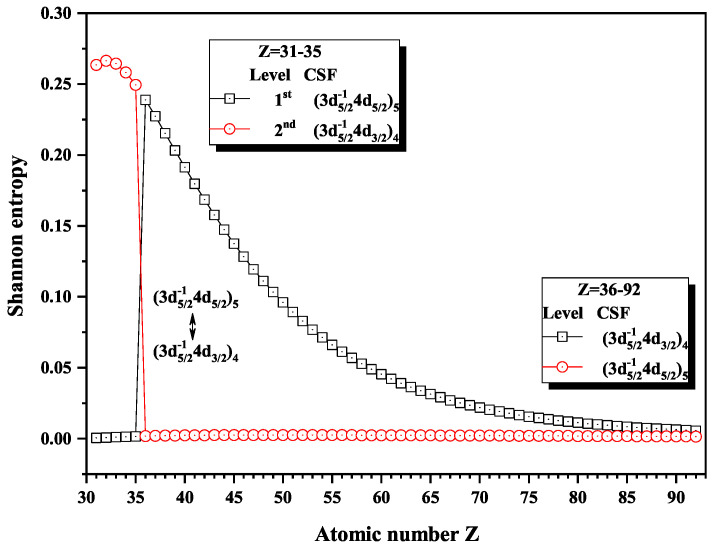
Shannon entropies for the 1st and 2nd levels labeled as (3d${}_{5/2}^{-1}$4d${}_{5/2}{)}_{5}$ and (3d${}_{5/2}^{-1}$4d${}_{3/2}{)}_{4}$ in the mixed subspace with $J^{P}=4^{e}$ and 5*^e^* for Ni-like isoelectronic sequence with Z = 31–92.

**Figure 9:**
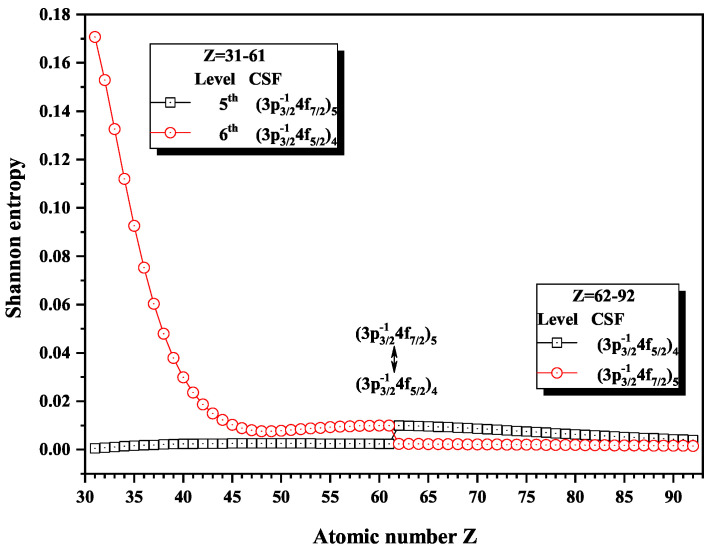
Shannon entropies for the 5th and 6th levels labeled as (3p${}_{3/2}^{-1}$4f${}_{7/2}{)}_{5}$ and (3p${}_{3/2}^{-1}$4f${}_{5/2}{)}_{4}$ in the mixed subspace with $J^{P}=4^{e}$ and 5*^e^* for Ni-like isoelectronic sequence with Z = 31–92.

**Figure 10:**
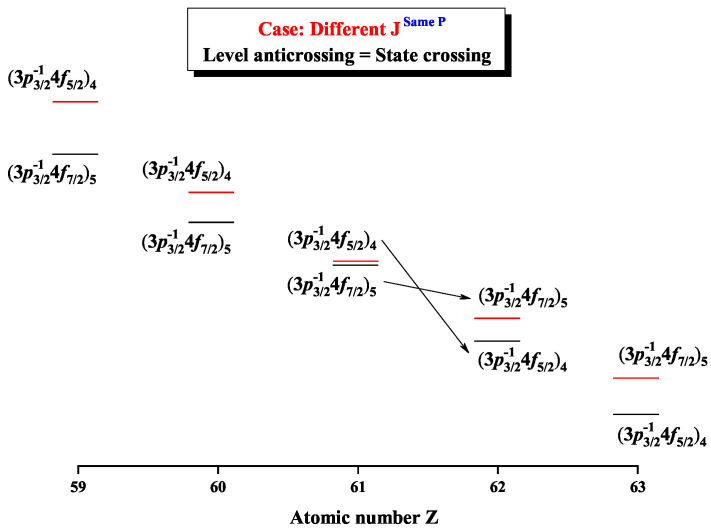
Energy diagrams for the 5th and 6th levels labeled as (3p${}_{3/2}^{-1}$4f${}_{7/2}{)}_{5}$ and (3p${}_{3/2}^{-1}$4f${}_{5/2}{)}_{4}$ in the mixed subspace with $J^{P}=4^{e}$ and 5*^e^* for Ni-like isoelectronic sequence with Z = 59–63.

Figure 8 and Figure 9 give the Shannon entropies for the 1st, 2nd, 5th, and 6th levels in the mixed subspace with $J^{P}=4^{e}$ and 5*^e^*. In Figure 8, the entropies jump at Z = 36, where the dominant components ${\left(3d_{5/2}^{-1}4d_{5/2}\right)}_{5}$ and ${\left(3d_{5/2}^{-1}4d_{3/2}\right)}_{4}$ exchange. Figure 9 shows that the entropies jump at Z = 62, where the dominant components ${\left(3p_{3/2}^{-1}4f_{7/2}\right)}_{5}$ and ${\left(3p_{3/2}^{-1}4f_{5/2}\right)}_{4}$ exchange with each other. In fact, the 5th and 6th levels, respectively, with $J^{P}=4^{e}$ and 5*^e^*, anticross at Z = 61, as shown in Figure 10. In other words, in combined subspace, the levels with the different *J* and the same *P*, which do not have configuration interaction, can also anticross besides those levels with the same $J^{P}$ which can interact with each other.

**Figure 11:**
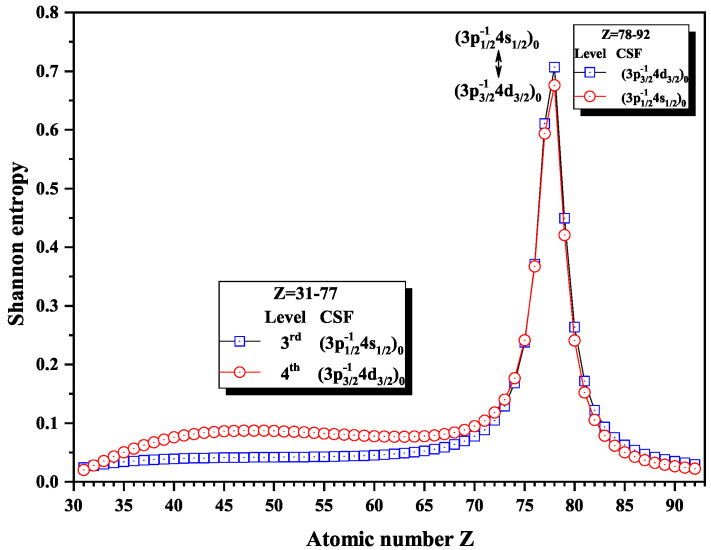
Shannon entropies for the 3rd and 4th levels labeled as (3p${}_{1/2}^{-1}$4s${}_{1/2}{)}_{0}$ and (3p${}_{3/2}^{-1}$4d${}_{3/2}{)}_{0}$ in the subspace with $J^{P}=0^{o}$ for Ni-like isoelectronic sequence with Z = 31–92.

Figure 11 gives the Shannon entropies for the 3rd and 4th levels in subspace with $J^{P}=0^{o}$. Both of them have the maxima at Z = 78, where the dominant components ${\left(3p_{1/2}^{-1}4s_{1/2}\right)}_{0}$ and ${\left(3p_{3/2}^{-1}4d_{3/2}\right)}_{0}$ exchange.

**Figure 12:**
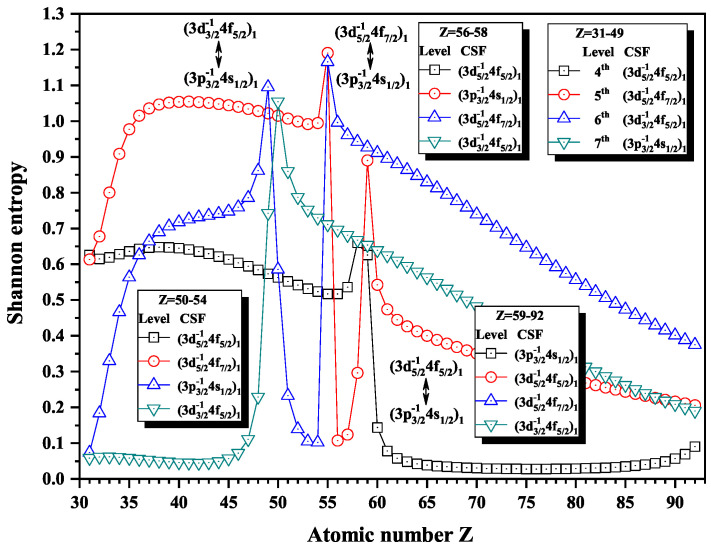
Shannon entropies for the 4th, 5th, 6th, and 7th levels labeled as (3d${}_{5/2}^{-1}$4f${}_{5/2}{)}_{1}$, (3d${}_{5/2}^{-1}$4f${}_{7/2}{)}_{1}$, (3d${}_{3/2}^{-1}$4f${}_{5/2}{)}_{1}$, and (3p${}_{3/2}^{-1}$4s${}_{1/2}{)}_{1}$ in the subspace with $J^{P}=1^{o}$ for Ni-like isoelectronic sequence with Z = 31–92.

**Figure 13:**
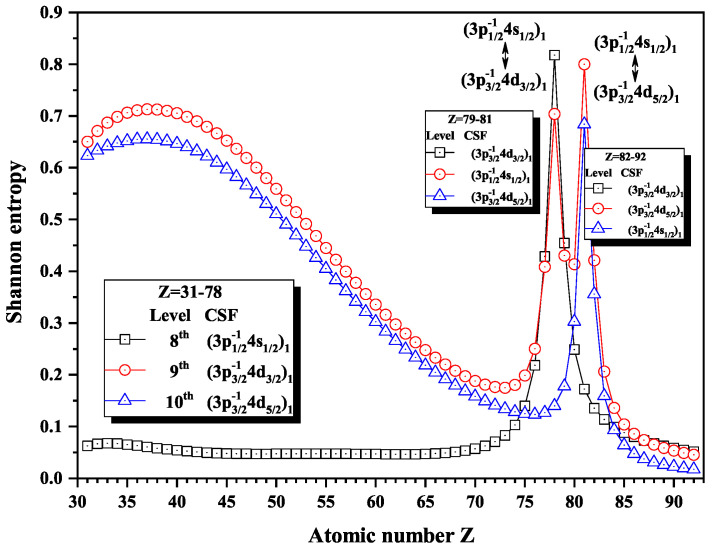
Shannon entropies for the 8th, 9th, and 10th levels labeled as (3p${}_{1/2}^{-1}$4s${}_{1/2}{)}_{1}$, (3p${}_{3/2}^{-1}$4d${}_{3/2}{)}_{1}$, and (3p${}_{3/2}^{-1}$4d${}_{5/2}{)}_{1}$ in the subspace with $J^{P}=1^{o}$ for Ni-like isoelectronic sequence with Z = 31–92.

**Figure 14:**
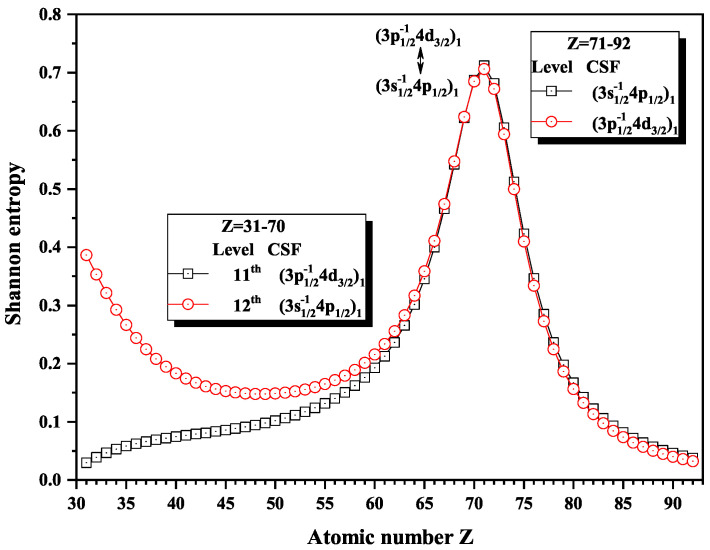
Shannon entropies for the 11th and 12th levels labeled as (3p${}_{3/2}^{-1}$4d${}_{3/2}{)}_{1}$ and (3s${}_{1/2}^{-1}$4p${}_{1/2}{)}_{1}$ in the subspace with $J^{P}=1^{o}$ for Ni-like isoelectronic sequence with Z = 31–92.

Figure 12, Figure 13 and Figure 14 give the Shannon entropies for the 4th, 5th, 6th, 7th, 8th, 9th, 10th, 11th, and 12th levels in subspace with $J^{P}=1^{o}$. In Figure 12, the entropies have two maxima at Z = 49 and 55 for the 6th level, one maximum at Z = 50 for the 7th level, two maxima at Z = 55 and 59 for the 5th level, and one maximum at Z = 58 for the 4th level. Meanwhile, the dominant components (3d${}_{3/2}^{-1}$4f${}_{5/2}{)}_{1}$, (3d${}_{5/2}^{-1}$4f${}_{7/2}{)}_{1}$, and (3d${}_{5/2}^{-1}$4f${}_{5/2}{)}_{1}$ exchange with (3p${}_{3/2}^{-1}$4s${}_{1/2}{)}_{1}$, in turn, at Z = 50, 56, and 59. In Figure 13, the entropies have one, two, and one maxima for 8th, 9th, and 10th levels at Z = 77 and 81, where the dominant components (3p${}_{1/2}^{-1}$4s${}_{1/2}{)}_{1}$ exchange with (3p${}_{3/2}^{-1}$4d${}_{3/2}{)}_{1}$ and (3p${}_{3/2}^{-1}$4d${}_{5/2}{)}_{1}$. In Figure 14, both the entropies of the 11th and 12th levels have maxima at Z = 71, where the dominant components (3p${}_{1/2}^{-1}$4d${}_{3/2}{)}_{1}$ and (3s${}_{1/2}^{-1}$4p${}_{1/2}{)}_{1}$ exchange.

**Figure 15:**
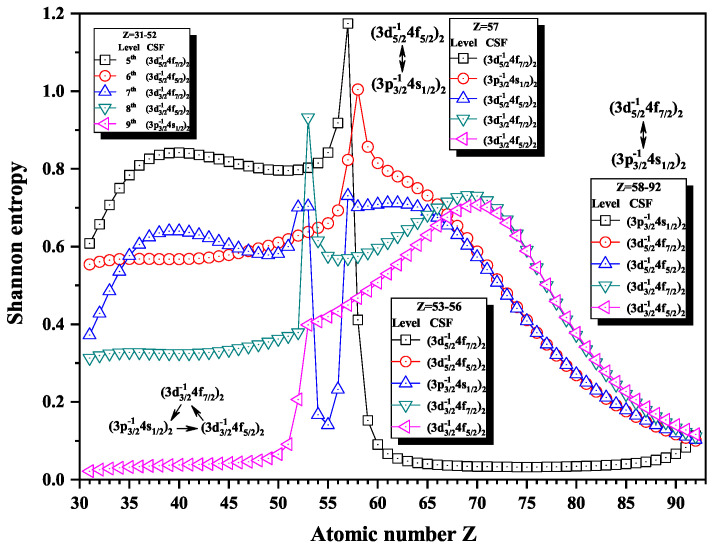
Shannon entropies for the 5th, 6th, 7th, 8th, and 9th levels labeled as (3d${}_{5/2}^{-1}$4f${}_{7/2}{)}_{2}$, (3d${}_{5/2}^{-1}$4f${}_{5/2}{)}_{2}$, (3d${}_{3/2}^{-1}$4f${}_{7/2}{)}_{2}$, (3d${}_{3/2}^{-1}$4f${}_{5/2}{)}_{2}$, and (3p${}_{3/2}^{-1}$4s${}_{1/2}{)}_{2}$ in the subspace with $J^{P}=2^{o}$ for Ni-like isoelectronic sequence with Z = 31–92.

Figure 15 gives the Shannon entropies for the 5th, 6th, 7th, 8th, and 9th levels in subspace with $J^{P}=2^{o}$. In Figure 15, the entropies of the 9th level jump at Z = 53, where the entropies of the 7th and 8th have maxima. It is interesting that the dominant components (3d${}_{5/2}^{-1}$4f${}_{7/2}{)}_{2}$, (3p${}_{3/2}^{-1}$4s${}_{1/2}{)}_{2}$, and (3d${}_{3/2}^{-1}$4f${}_{5/2}{)}_{2}$ form triangle exchange at Z = 53. There are maxima at Z = 57 for the 5th and 7th levels and at Z = 58 for the 6th level. Meanwhile, the dominant components (3d${}_{5/2}^{-1}$4f${}_{5/2}{)}_{2}$ and (3d${}_{5/2}^{-1}$4f${}_{7/2}{)}_{2}$ exchange with (3p${}_{3/2}^{-1}$4s${}_{1/2}{)}_{2}$, in turn, at Z = 57 and 58.

**Figure 16:**
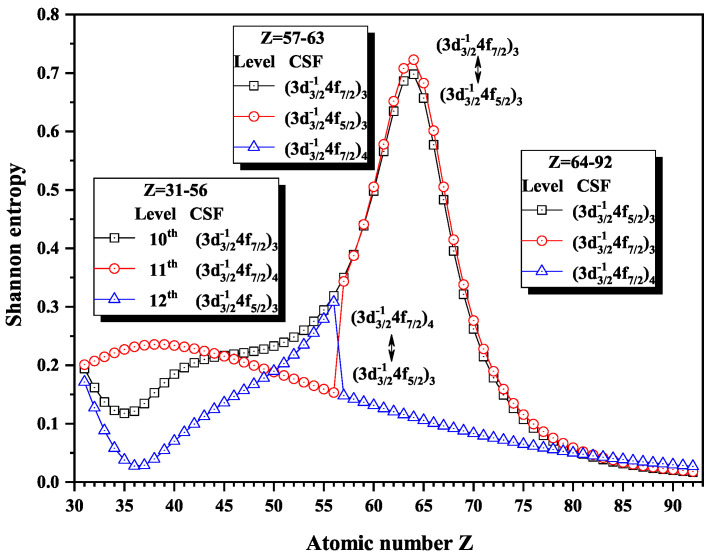
Shannon entropies for the 10th, 11th, and 12th levels labeled as (3d${}_{3/2}^{-1}$4f${}_{7/2}{)}_{3}$, (3d${}_{3/2}^{-1}$4f${}_{7/2}{)}_{4}$, and (3d${}_{3/2}^{-1}$4f${}_{5/2}{)}_{3}$ in the mixed subspace with $J^{P}=3^{o}$ and $4^{o}$ for Ni-like isoelectronic sequence with Z = 31–92.

**Figure 17:**
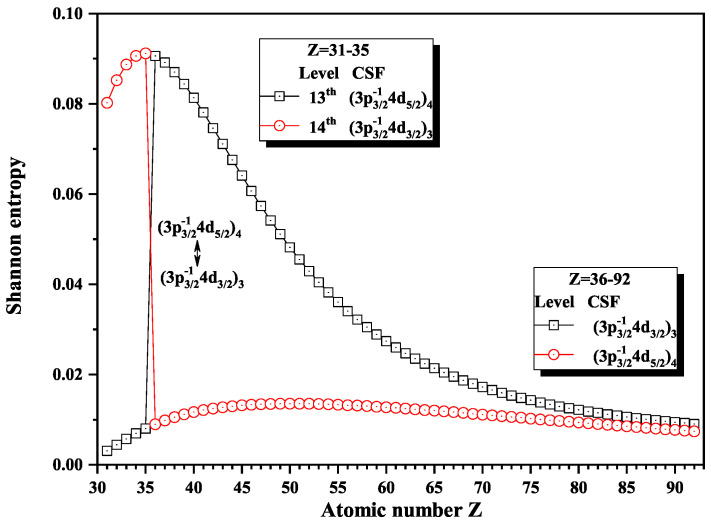
Shannon entropies for the 13th and 14th levels labeled as (3p${}_{3/2}^{-1}$4d${}_{5/2}{)}_{4}$ and (3p${}_{3/2}^{-1}$4p${}_{3/2}{)}_{3}$ in the mixed subspace with $J^{P}=3^{o}$ and $4^{o}$ for Ni-like isoelectronic sequence with Z = 31–92.

**Figure 18:**
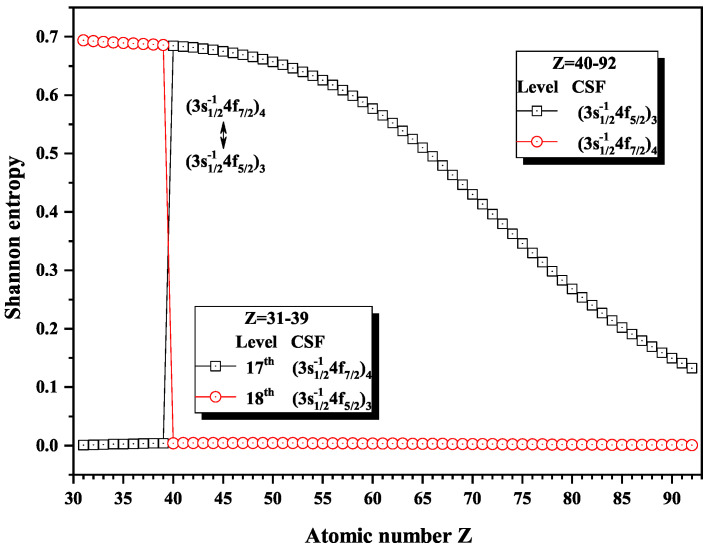
Shannon entropies for the 17th and 18th levels labeled as (3s${}_{1/2}^{-1}$4f${}_{7/2}{)}_{4}$ and (3s${}_{1/2}^{-1}$4f${}_{5/2}{)}_{3}$ in the mixed subspace with $J^{P}=3^{o}$ and $4^{o}$ for Ni-like isoelectronic sequence with Z = 31–92.

Figure 16, Figure 17 and Figure 18 give the Shannon entropies for the 10th, 11th, 12th, 13th, 14th, 17th, and 18th levels in the mixed subspace with $J^{P}=3^{o}$ and $4^{o}$. In Figure 16, the entropies of the 11th and 12th levels jump at Z = 57, where the dominant components (3d${}_{3/2}^{-1}$4f${}_{7/2}{)}_{4}$ and (3d${}_{3/2}^{-1}$4f${}_{5/2}{)}_{3}$ exchange. At Z = 64, the entropies have maxima corresponding to the exchange between the dominant components (3d${}_{3/2}^{-1}$4f${}_{7/2}{)}_{3}$ and (3d${}_{3/2}^{-1}$4f${}_{5/2}{)}_{3}$ for the 10th and 11th levels. Figure 17 shows that the entropies of the 13th and 14th levels jump at Z = 36, where the dominant components (3p${}_{3/2}^{-1}$4d${}_{5/2}{)}_{4}$ and (3p${}_{3/2}^{-1}$4p${}_{3/2}{)}_{3}$ exchange. Similarly, Figure 18 presents the entropies of the 17th and 18th levels jump at Z = 40, where the dominant components (3s${}_{1/2}^{-1}$4f${}_{7/2}{)}_{4}$ and (3s${}_{1/2}^{-1}$4f${}_{5/2}{)}_{3}$ exchange.

In order to better show the connection between the sudden change of Shannon entropies, information exchanges, eigenlevel anticrossings, and strong configuration interactions, all of them have been collected in Table 6, Table 7, Table 8, Table 9, Table 10, Table 11, Table 12, Table 13 and Table 14. In these tables, the sudden change is labeled as “Yes” or “No” and the eigenlevel anticrossings are described by the minima of the energy difference between the two corresponding levels; otherwise, “No” is also used. In addition to the sudden change and the minima of the energy differences, the associated levels are illustrated by the atomic state functions which have at most three CSF components, and the coefficients are written in a bold font for the dominant CSFs. It is found that, where there is a sudden change in Shannon entropy, there is an eigenlevel anticrossing, and vice versa. Although there is a minimum $\Delta E_{19,16}=1.62763$ in the subspace with $J^{P}=2^{e}$ and 3*^e^* at Z = 39, this minimum is not so obvious that we can think that it does not affect the general law because the energy differences are $\Delta E_{19,16}=1.62922$ and $\Delta E_{19,16}=1.62774$ at Z = 38 and 40, respectively. At the same time, some sufficient conditions could be obtained. That is, if there is a sudden change in Shannon entropy, then the information exchange can take place, and if there is an eigenlevel anticrossing, there is an information exchange. Moreover, it is also clarified that there is no necessary connection between strong configuration interaction and eigenlevel anticrossing and information exchange. Because the eigenlevel anticrossing can be explained by the energy difference which should not depend on the configuration basis in different coupling schemes that, however, the (strong) configuration interaction relies on.

## 4. Summary and Outlook

Based on the wavefunctions calculated by using the multiconfiguration Dirac–Hartree–Fock (MCDHF) and relativistic configuration interaction (RCI) methods, the Shannon entropies have been obtained for the ground and excited states of Ni-like isoelectronic sequence. The role of Shannon entropy can be considered as an information measurement of atomic state in configuration space. The larger the entropy, the more ambiguous the information of the energy level and the less meaningful the configuration is. In the present work, a relationship was found among the sudden change of Shannon entropy, information exchange, eigenlevel anticrossing, and strong configuration interaction.

Firstly, the sudden change of Shannon entropy is a sufficient and necessary condition for the eigenlevel anticrossing in a given configuration space, which means that the sudden change of Shannon entropy can be considered as the effective indicator of the eigenlevel anticrossing, likely in the study on the one-electron atom in an external field [2,3]. In fact, the eigenlevel anticrossing always occurs near the sudden change of entropy, rather than at the exact location of the sudden change, because the discreteness in the isoelectronic sequence. Secondly, if there are sudden changes of Shannon entropy and eigenlevel anticrossings, information exchange must take place, which is very much the same in the study on one-electron atom in an external field. However, there is no sudden change of entropy and eigenlevel anticrossing for individual information exchanges. So, the sudden change in Shannon entropy and eigenlevel anticrossings could be considered as a sufficient condition for information exchanges. Certainly, not only the levels with the same $J^{P}$ but also those with different symmetry can anticross and exchange their information in a given configuration space, along with isoelectronic sequence. The levels with same $J^{P}$ exchanging their information in a configuration space could be reflected by the sharp peak and jump in Shannon entropy as the increasing of nuclear charge Z, while eigenlevel anticrossings and information exchanges between those with different $J^{P}$ can only be shown by the jump in Shannon entropy, along with the isoelectronic sequence.

In addition, it was previously thought that there was an inevitable relationship between the eigenlevel anticrossing and strong configuration interaction in isoelectronic sequences. However, according to the present analysis, there is no necessary causal relationship between them. In theory, the calculated results of energy levels should be the same regardless of the coupling basis set used, but the strength of configuration interaction is related to the coupling mechanism. In fact, the strength of configuration interaction is usually reflected by configuration mixing coefficients. When each configuration mixing coefficient approaches the average $n_{c}^{-1}$ in a large enough configuration space, which means the entropy of the corresponding level approaches the maximum ln$n_{c}$, configuration interaction is the strongest. On the contrary, if the configuration mixing coefficients nearly form a unit coordinate vector, then the configuration interaction becomes the weakest. Very commonly for simple level structure in atomic systems, the configuration interaction is strong in the jj coupling scheme but weak in the LS coupling scheme, and vice versa. Of course, configuration interaction, all of which are strong in the jj and LS coupling scheme, may be very weak in other coupling schemes as is more common in the description of doubly excited states [32,33], especially for the doubly Rydberg states [34] similar to the quantum chaotic system mentioned by [4,5]. Therefore, we have the opportunity to rethink the relationship between strong configuration interaction and eigenlevel anticrossing; that is, strong configuration interaction and eigenlevel anticrossing do not always occur at the same time.

Of course, it is hoped that a rigorous theory about Shannon entropy in configuration space is required in order to help us to better understand the traditional atomic physics in various isoelectronic sequences. However, there is no analytical expression in the study of isoelectronic sequences, unlike in the calculation of the entropy of one-electron atom in an external field [2,3]; therefore, a number of numerical calculations should be indispensable even if the quantum chaotic systems have been successfully analyzed by using the combination of the perturbation theory and statistical theory [4,5]. In addition, inspired by nuclear physics [5,35], we also hope to give a new expression to the uncertainty relation.

## Figures and Tables

**Table 1:** Subspaces expanded by the ground and single excited configuration state functions with $J^{P}=0^{e},1^{e},2^{e},3^{e},4^{e}$, and 5*^e^*.

0*^e^*	1*^e^*	2*^e^*	3*^e^*	4*^e^*	5*^e^*
(3d${}_{3/2}^{4}$3d${}_{5/2}^{6}{)}_{0}$	(3d${}_{5/2}^{-1}$4d${}_{3/2}{)}_{1}$	(3d${}_{5/2}^{-1}$4s${}_{1/2}{)}_{2}$	(3d${}_{5/2}^{-1}$4s${}_{1/2}{)}_{3}$	(3d${}_{5/2}^{-1}$4d${}_{3/2}{)}_{4}$	(3d${}_{5/2}^{-1}$4d${}_{5/2}{)}_{5}$
(3d${}_{5/2}^{-1}$4d${}_{5/2}{)}_{0}$	(3d${}_{5/2}^{-1}$4d${}_{5/2}{)}_{1}$	(3d${}_{5/2}^{-1}$4d${}_{3/2}{)}_{2}$	(3d${}_{5/2}^{-1}$4d${}_{3/2}{)}_{3}$	(3d${}_{5/2}^{-1}$4d${}_{5/2}{)}_{4}$	(3p${}_{3/2}^{-1}$4f${}_{7/2}{)}_{5}$
(3d${}_{3/2}^{-1}$4d${}_{3/2}{)}_{0}$	(3d${}_{3/2}^{-1}$4s${}_{1/2}{)}_{1}$	(3d${}_{5/2}^{-1}$4d${}_{5/2}{)}_{2}$	(3d${}_{5/2}^{-1}$4d${}_{5/2}{)}_{3}$	(3d${}_{3/2}^{-1}$4d${}_{5/2}{)}_{4}$	
(3p${}_{3/2}^{-1}$4p${}_{3/2}{)}_{0}$	(3d${}_{3/2}^{-1}$4d${}_{3/2}{)}_{1}$	(3d${}_{3/2}^{-1}$4s${}_{1/2}{)}_{2}$	(3d${}_{3/2}^{-1}$4d${}_{3/2}{)}_{3}$	(3p${}_{3/2}^{-1}$4f${}_{5/2}{)}_{4}$	
(3p${}_{1/2}^{-1}$4p${}_{1/2}{)}_{0}$	(3d${}_{3/2}^{-1}$4d${}_{5/2}{)}_{1}$	(3d${}_{3/2}^{-1}$4d${}_{3/2}{)}_{2}$	(3d${}_{3/2}^{-1}$4d${}_{5/2}{)}_{3}$	(3p${}_{3/2}^{-1}$4f${}_{7/2}{)}_{4}$	
(3s${}_{1/2}^{-1}$4s${}_{1/2}{)}_{0}$	(3p${}_{3/2}^{-1}$4p${}_{1/2}{)}_{1}$	(3d${}_{3/2}^{-1}$4d${}_{5/2}{)}_{2}$	(3p${}_{3/2}^{-1}$4p${}_{3/2}{)}_{3}$	(3p${}_{1/2}^{-1}$4f${}_{7/2}{)}_{4}$	
	(3p${}_{3/2}^{-1}$4p${}_{3/2}{)}_{1}$	(3p${}_{3/2}^{-1}$4p${}_{1/2}{)}_{2}$	(3p${}_{3/2}^{-1}$4f${}_{5/2}{)}_{3}$		
	(3p${}_{3/2}^{-1}$4f${}_{5/2}{)}_{1}$	(3p${}_{3/2}^{-1}$4p${}_{3/2}{)}_{2}$	(3p${}_{3/2}^{-1}$4f${}_{7/2}{)}_{3}$		
	(3p${}_{1/2}^{-1}$4p${}_{1/2}{)}_{1}$	(3p${}_{3/2}^{-1}$4f${}_{5/2}{)}_{2}$	(3p${}_{1/2}^{-1}$4f${}_{5/2}{)}_{3}$		
	(3p${}_{1/2}^{-1}$4p${}_{3/2}{)}_{1}$	(3p${}_{3/2}^{-1}$4f${}_{7/2}{)}_{2}$	(3p${}_{1/2}^{-1}$4f${}_{7/2}{)}_{3}$		
	(3s${}_{1/2}^{-1}$4s${}_{1/2}{)}_{1}$	(3p${}_{1/2}^{-1}$4p${}_{3/2}{)}_{2}$	(3s${}_{1/2}^{-1}$4d${}_{5/2}{)}_{3}$		
	(3s${}_{1/2}^{-1}$4d${}_{3/2}{)}_{1}$	(3p${}_{1/2}^{-1}$4f${}_{5/2}{)}_{2}$			
		(3s${}_{1/2}^{-1}$4d${}_{3/2}{)}_{2}$			
		(3s${}_{1/2}^{-1}$4d${}_{5/2}{)}_{2}$			
					
$n_{c}\left(0^{e}\right)=6$	$n_{c}\left(1^{e}\right)=12$	$n_{c}\left(2^{e}\right)=14$	$n_{c}\left(3^{e}\right)=11$	$n_{c}\left(4^{e}\right)=6$	$n_{c}\left(5^{e}\right)=2$

**Table 2:** Subspaces expanded by the single excited configuration state functions with $J^{P}\;=\;0^{o},1^{o},2^{o},3^{o},4^{o},5^{o}$, and $6^{o}$.

0${}^{o}$	1${}^{o}$	2${}^{o}$	3${}^{o}$	4${}^{o}$	5${}^{o}$	6${}^{o}$
(3d${}_{5/2}^{-1}$4f${}_{5/2}{)}_{0}$	(3d${}_{5/2}^{-1}$4p${}_{3/2}{)}_{1}$	(3d${}_{5/2}^{-1}$4p${}_{1/2}{)}_{2}$	(3d${}_{5/2}^{-1}$4p${}_{1/2}{)}_{3}$	(3d${}_{5/2}^{-1}$4p${}_{3/2}{)}_{4}$	(3d${}_{5/2}^{-1}$4f${}_{5/2}{)}_{5}$	(3d${}_{5/2}^{-1}$4f${}_{7/2}{)}_{6}$
(3d${}_{3/2}^{-1}$4p${}_{3/2}{)}_{0}$	(3d${}_{5/2}^{-1}$4f${}_{5/2}{)}_{1}$	(3d${}_{5/2}^{-1}$4p${}_{3/2}{)}_{2}$	(3d${}_{5/2}^{-1}$4p${}_{3/2}{)}_{3}$	(3d${}_{5/2}^{-1}$4f${}_{5/2}{)}_{4}$	(3d${}_{5/2}^{-1}$4f${}_{7/2}{)}_{5}$	
(3p${}_{3/2}^{-1}$4d${}_{3/2}{)}_{0}$	(3d${}_{5/2}^{-1}$4f${}_{7/2}{)}_{1}$	(3d${}_{5/2}^{-1}$4f${}_{5/2}{)}_{2}$	(3d${}_{5/2}^{-1}$4f${}_{5/2}{)}_{3}$	(3d${}_{5/2}^{-1}$4f${}_{7/2}{)}_{4}$	(3d${}_{3/2}^{-1}$4f${}_{7/2}{)}_{5}$	
(3p${}_{1/2}^{-1}$4s${}_{1/2}{)}_{0}$	(3d${}_{3/2}^{-1}$4p${}_{1/2}{)}_{1}$	(3d${}_{5/2}^{-1}$4f${}_{7/2}{)}_{2}$	(3d${}_{5/2}^{-1}$4f${}_{7/2}{)}_{3}$	(3d${}_{3/2}^{-1}$4f${}_{5/2}{)}_{4}$		
(3s${}_{1/2}^{-1}$4p${}_{1/2}{)}_{0}$	(3d${}_{3/2}^{-1}$4p${}_{3/2}{)}_{1}$	(3d${}_{3/2}^{-1}$4p${}_{1/2}{)}_{2}$	(3d${}_{3/2}^{-1}$4p${}_{3/2}{)}_{3}$	(3d${}_{3/2}^{-1}$4f${}_{7/2}{)}_{4}$		
	(3d${}_{3/2}^{-1}$4f${}_{5/2}{)}_{1}$	(3d${}_{3/2}^{-1}$4p${}_{3/2}{)}_{2}$	(3d${}_{3/2}^{-1}$4f${}_{5/2}{)}_{3}$	(3p${}_{3/2}^{-1}$4d${}_{5/2}{)}_{4}$		
	(3p${}_{3/2}^{-1}$4s${}_{1/2}{)}_{1}$	(3d${}_{3/2}^{-1}$4f${}_{5/2}{)}_{2}$	(3d${}_{3/2}^{-1}$4f${}_{7/2}{)}_{3}$	(3s${}_{1/2}^{-1}$4f${}_{7/2}{)}_{4}$		
	(3p${}_{3/2}^{-1}$4d${}_{3/2}{)}_{1}$	(3d${}_{3/2}^{-1}$4f${}_{7/2}{)}_{2}$	(3p${}_{3/2}^{-1}$4d${}_{3/2}{)}_{3}$			
	(3p${}_{3/2}^{-1}$4d${}_{5/2}{)}_{1}$	(3p${}_{3/2}^{-1}$4s${}_{1/2}{)}_{2}$	(3p${}_{3/2}^{-1}$4d${}_{5/2}{)}_{3}$			
	(3p${}_{1/2}^{-1}$4s${}_{1/2}{)}_{1}$	(3p${}_{3/2}^{-1}$4d${}_{3/2}{)}_{2}$	(3p${}_{1/2}^{-1}$4d${}_{5/2}{)}_{3}$			
	(3p${}_{1/2}^{-1}$4d${}_{3/2}{)}_{1}$	(3p${}_{3/2}^{-1}$4d${}_{5/2}{)}_{2}$	(3s${}_{1/2}^{-1}$4f${}_{5/2}{)}_{3}$			
	(3s${}_{1/2}^{-1}$4p${}_{1/2}{)}_{1}$	(3p${}_{1/2}^{-1}$4d${}_{3/2}{)}_{2}$	(3s${}_{1/2}^{-1}$4f${}_{7/2}{)}_{3}$			
	(3s${}_{1/2}^{-1}$4p${}_{3/2}{)}_{1}$	(3p${}_{1/2}^{-1}$4d${}_{5/2}{)}_{2}$				
		(3s${}_{1/2}^{-1}$4p${}_{3/2}{)}_{2}$				
		(3s${}_{1/2}^{-1}$4f${}_{5/2}{)}_{2}$				
						
$n_{c}\left(0^{o}\right)=5$	$n_{c}\left(1^{o}\right)=13$	$n_{c}\left(2^{o}\right)=15$	$n_{c}\left(3^{o}\right)=12$	$n_{c}\left(4^{o}\right)=7$	$n_{c}\left(5^{o}\right)=3$	$n_{c}\left(6^{o}\right)=1$

**Table 3:** Configuration mixing coefficients for the 4th, 5th, and 6th levels in the subspace with $J^{P}=1^{e}$ at Z = 41, 42, 43, 87, 88, 91, and 92.

CSF	Z =41				Z =42				Z =43				Z =87				Z =88				Z =91				Z =92		
	4th	5th	6th		4th	5th	6th		4th	5th	6th		4th	5th	6th		4th	5th	6th		4th	5th	6th		4th	5th	6th
(3d${}_{5/2}^{-1}$4d${}_{3/2}{)}_{1}$	0.2286	0.0020	−0.0518		0.2186	−0.0065	−0.0532		0.2085	0.0145	−0.0545		−0.0282	−0.0697	−0.0674		−0.0300	−0.0987	0.0082		−0.0838	−0.0759	−0.0100		−0.1157	−0.0192	−0.0108
(3d${}_{5/2}^{-1}$4d${}_{5/2}{)}_{1}$	−0.2293	0.1266	0.0265		−0.2178	0.1264	0.0258		−0.2060	−0.1262	0.0250		0.0170	0.0340	−0.0014		0.0170	0.0255	0.0217		0.0263	0.0117	0.0236		0.0297	−0.0038	0.0230
(3d${}_{3/2}^{-1}$4s${}_{1/2}{)}_{1}$	−0.0057	−0.0208	−0.0047		−0.0063	−0.0203	−0.0050		−0.0069	0.0197	−0.0053		0.0078	−0.0081	−0.0041		0.0075	−0.0090	−0.0019		0.0014	−0.0108	−0.0034		−0.0047	−0.0096	−0.0034
(3d${}_{3/2}^{-1}$4d${}_{3/2}{)}_{1}$	−0.6570	**−0.7333**	−0.0132		**−0.6829**	**−0.7108**	−0.0134		**−0.7092**	0.6862	−0.0137		0.9945	−0.0932	−0.0351		0.9924	−0.1161	−0.0259		**0.7755**	−0.6294	−0.0386		0.3029	**−0.9518**	−0.0380
(3d${}_{3/2}^{-1}$4d${}_{5/2}{)}_{1}$	**0.6796**	−0.6669	0.0198		0.6609	−0.6907	0.0197		0.6399	**0.7151**	0.0196		−0.0554	**−0.8161**	0.5734		−0.0544	−0.2570	**−0.9638**		−0.0661	−0.0214	−0.9967		−0.0544	0.0218	−0.9974
(3p${}_{3/2}^{-1}$4p${}_{1/2}{)}_{1}$	−0.0009	−0.0030	0.9030		−0.0018	−0.0029	0.9158		−0.0026	0.0028	0.9271		0.0794	0.5638	**0.8156**		0.1025	**0.9534**	−0.2633		0.6212	**0.7726**	−0.0609		**0.9436**	0.3046	−0.0479
(3p${}_{3/2}^{-1}$4p${}_{3/2}{)}_{1}$	−0.0030	−0.0028	−0.4013		−0.0025	−0.0032	−0.3740		−0.0020	0.0036	−0.3476		−0.0040	−0.0275	−0.0048		−0.0044	−0.0231	−0.0145		−0.0125	−0.0119	−0.0200		−0.0158	−0.0027	−0.0206
(3p${}_{3/2}^{-1}$4f${}_{5/2}{)}_{1}$	−0.0015	−0.0055	−0.0026		−0.0017	−0.0055	−0.0027		−0.0020	0.0055	−0.0028		0.0050	−0.0038	−0.0006		0.0049	−0.0034	−0.0020		0.0026	−0.0050	−0.0026		−0.0005	−0.0056	−0.0026
(3p${}_{1/2}^{-1}$4p${}_{1/2}{)}_{1}$	−0.0213	−0.0252	0.0278		−0.0226	−0.0249	0.0244		−0.0238	0.0244	0.0214		0.0205	−0.0018	−0.0006		0.0200	−0.0022	−0.0005		0.0146	−0.0118	−0.0007		0.0056	−0.0174	−0.0007
(3p${}_{1/2}^{-1}$4p${}_{3/2}{)}_{1}$	0.0319	−0.0205	−0.1090		0.0319	−0.0219	−0.1009		0.0316	0.0233	−0.0932		−0.0053	−0.0183	0.0059		−0.0052	−0.0097	−0.0158		−0.0068	−0.0021	−0.0161		−0.0063	0.0020	−0.0156
(3s${}_{1/2}^{-1}$4s${}_{1/2}{)}_{1}$	0.0063	−0.0021	−0.0828		0.0066	−0.0024	−0.0813		0.0069	0.0028	−0.0795		−0.0051	−0.0157	−0.0098		−0.0054	−0.0176	−0.0027		−0.0122	−0.0098	−0.0061		−0.0150	−0.0013	−0.0062
(3s${}_{1/2}^{-1}$4d${}_{3/2}{)}_{1}$	−0.0015	−0.0055	−0.0052		−0.0017	−0.0056	−0.0056		−0.0019	0.0056	−0.0059		0.0030	−0.0036	−0.0020		0.0029	−0.0040	−0.0007		0.0004	−0.0044	−0.0014		−0.0020	−0.0038	−0.0014

**Table 4:** Configuration mixing coefficients for the 8th, 9th, 10th, and 11th levels in the subspace with $J^{P}=1^{e}$ at Z = 37, 38, 69, and 70.

CSF	Z =37					Z =38					Z =69					Z =70			
	8th	9th	10th	11th		8th	9th	10th	11th		8th	9th	10th	11th		8th	9th	10th	11th
(3d${}_{5/2}^{-1}$4d${}_{3/2}{)}_{1}$	−0.0008	−0.0009	0.0020	−0.0060		−0.0008	−0.0013	−0.0063	−0.0021		0.0002	−0.0055	−0.0064	−0.0026		0.0001	−0.0055	−0.0022	−0.0064
(3d${}_{5/2}^{-1}$4d${}_{5/2}{)}_{1}$	−0.0001	−0.0002	0.0021	0.0057		−0.0003	0.0001	0.0059	−0.0022		0.0037	0.0041	0.0062	−0.0025		0.0037	0.0041	−0.0028	0.0060
(3d${}_{3/2}^{-1}$4s${}_{1/2}{)}_{1}$	−0.0204	−0.0065	0.0014	0.0000		−0.0205	−0.0066	0.0000	−0.0015		0.0152	−0.0048	0.0004	−0.0008		0.0149	−0.0047	−0.0007	0.0004
(3d${}_{3/2}^{-1}$4d${}_{3/2}{)}_{1}$	0.0301	−0.0083	−0.0039	−0.0029		0.0314	−0.0085	−0.0031	0.0042		−0.0299	−0.0067	−0.0027	0.0051		−0.0294	−0.0066	0.0052	−0.0023
(3d${}_{3/2}^{-1}$4d${}_{5/2}{)}_{1}$	0.0026	0.0358	−0.0020	0.0059		0.0024	0.0373	0.0061	0.0021		−0.0001	0.0314	0.0040	0.0024		−0.0001	0.0308	0.0022	0.0037
(3p${}_{3/2}^{-1}$4p${}_{1/2}{)}_{1}$	−0.0146	−0.1078	0.0049	−0.0527		−0.0128	−0.1020	−0.0535	−0.0050		−0.0004	−0.0204	−0.0285	−0.0035		−0.0004	−0.0199	−0.0021	−0.0275
(3p${}_{3/2}^{-1}$4p${}_{3/2}{)}_{1}$	−0.0993	0.1136	0.0044	0.0600		−0.0962	0.1084	0.0612	−0.0045		0.0206	0.0253	0.0400	−0.0023		0.0196	0.0247	−0.0041	0.0386
(3p${}_{3/2}^{-1}$4f${}_{5/2}{)}_{1}$	−0.0145	−0.0047	**0.9987**	−0.0003		−0.0146	−0.0048	0.0000	**−0.9985**		0.0210	−0.0123	0.0156	**−0.9989**		0.0220	−0.0143	**−0.9985**	−0.0315
(3p${}_{1/2}^{-1}$4p${}_{1/2}{)}_{1}$	−0.9929	0.0300	−0.0162	−0.0219		−0.9934	0.0251	−0.0226	0.0165		0.9983	−0.0062	−0.0285	0.0217		0.9983	−0.0064	0.0240	−0.0273
(3p${}_{1/2}^{-1}$4p${}_{3/2}{)}_{1}$	−0.0406	−0.9852	−0.0057	0.0633		−0.0351	−0.9863	0.0657	0.0058		−0.0012	−0.9835	0.1755	0.0152		−0.0012	−0.9813	0.0085	0.1884
(3s${}_{1/2}^{-1}$4s${}_{1/2}{)}_{1}$	0.0140	−0.0505	−0.0003	**−0.9945**		0.0149	−0.0533	**−0.9942**	0.0000		−0.0278	−0.1739	**−0.9827**	−0.0138		−0.0279	−0.1863	0.0330	**−0.9800**
(3s${}_{1/2}^{-1}$4d${}_{3/2}{)}_{1}$	−0.0265	−0.0087	0.0468	0.0000		−0.0270	−0.0090	0.0000	−0.0508		0.0247	−0.0101	0.0015	−0.0356		0.0245	−0.0101	−0.0342	0.0000

**Table 5:** Configuration mixing coefficients for the 8th, 9th, 10th, and 11th levels in the subspace with $J^{P}=1^{e}$ at Z = 74, 75, 80, 81, 85, and 86.

CSF	Z =74					Z =75					Z =80					Z =81					Z =85					Z =86			
	8th	9th	10th	11th		8th	9th	10th	11th		8th	9th	10th	11th		8th	9th	10th	11th		8th	9th	10th	11th		8th	9th	10th	11th
(3d${}_{5/2}^{-1}$4d${}_{3/2}{)}_{1}$	0.0001	−0.0057	0.0021	−0.0056		0.0001	−0.0055	0.0027	0.0054		0.0001	−0.0024	−0.0066	0.0026		0.0001	−0.0024	−0.0068	0.0016		0.0008	−0.0022	−0.0064	−0.0010		−0.0020	−0.0011	−0.0062	−0.0012
(3d${}_{5/2}^{-1}$4d${}_{5/2}{)}_{1}$	0.0036	0.0042	0.0028	0.0055		0.0035	0.0010	−0.0050	−0.0053		0.0033	−0.0023	0.0058	−0.0030		0.0033	−0.0022	0.0060	−0.0021		0.0036	−0.0012	0.0060	0.0003		−0.0034	0.0015	0.0059	0.0006
(3d${}_{3/2}^{-1}$4s${}_{1/2}{)}_{1}$	0.0139	−0.0043	0.0004	0.0007		0.0137	−0.0032	0.0027	−0.0009		0.0124	−0.0004	−0.0030	−0.0022		0.0122	−0.0003	−0.0026	−0.0025		0.0108	0.0030	−0.0013	−0.0030		−0.0059	0.0093	−0.0011	−0.0030
(3d${}_{3/2}^{-1}$4d${}_{3/2}{)}_{1}$	−0.0274	−0.0059	−0.0053	−0.0019		−0.0269	−0.0004	0.0078	0.0017		−0.0245	0.0042	−0.0057	−0.0007		−0.0240	0.0040	−0.0054	−0.0014		−0.0223	−0.0026	−0.0041	−0.0030		0.0145	−0.0165	−0.0039	−0.0031
(3d${}_{3/2}^{-1}$4d${}_{5/2}{)}_{1}$	−0.0001	0.0283	−0.0009	0.0014		−0.0001	0.0200	−0.0191	−0.0006		−0.0001	0.0029	0.0225	0.0088		−0.0001	0.0029	0.0205	0.0116		−0.0009	0.0026	0.0132	0.0166		0.0024	0.0013	0.0121	0.0167
(3p${}_{3/2}^{-1}$4p${}_{1/2}{)}_{1}$	−0.0004	−0.0186	0.0018	−0.0229		−0.0003	−0.0143	0.0121	0.0216		−0.0003	−0.0030	−0.0211	0.0109		−0.0002	−0.0029	−0.0217	0.0076		0.0006	−0.0026	−0.0200	−0.0009		−0.0022	−0.0015	−0.0192	−0.0017
(3p${}_{3/2}^{-1}$4p${}_{3/2}{)}_{1}$	0.0163	0.0235	0.0040	0.0332		0.0156	0.0138	−0.0197	−0.0316		0.0125	−0.0017	0.0292	−0.0174		0.0120	−0.0016	0.0304	−0.0128		0.0101	0.0014	0.0291	−0.0005		−0.0063	0.0074	0.0282	0.0008
(3p${}_{3/2}^{-1}$4f${}_{5/2}{)}_{1}$	0.0276	−0.0641	**0.9971**	−0.0042		0.0297	**−0.7579**	−0.6509	0.0040		0.0516	−0.9983	0.0093	0.0048		0.0617	−0.9978	0.0069	0.0050		0.3519	**−0.9358**	0.0023	0.0039		**−0.8901**	−0.4552	0.0019	0.0035
(3p${}_{1/2}^{-1}$4p${}_{1/2}{)}_{1}$	0.9983	−0.0071	−0.0291	−0.0274		0.9983	0.0168	0.0274	0.0270		0.9976	0.0522	−0.0201	0.0207		0.9970	0.0623	−0.0228	0.0177		**0.9350**	0.3521	−0.0276	0.0076		−0.4545	**0.8897**	−0.0279	0.0062
(3p${}_{1/2}^{-1}$4p${}_{3/2}{)}_{1}$	−0.0011	**−0.9609**	−0.0610	0.2677		−0.0011	−0.6217	**0.7225**	−0.3007		−0.0009	−0.0100	**−0.7513**	−0.6593		−0.0009	−0.0081	−0.6494	**−0.7599**		0.0004	−0.0043	−0.3011	−0.9532		−0.0028	−0.0027	−0.2537	−0.9670
(3s${}_{1/2}^{-1}$4s${}_{1/2}{)}_{1}$	−0.0281	−0.2655	−0.0204	−0.9622		−0.0282	−0.1928	0.2290	0.9525		−0.0282	−0.0040	−0.6581	**0.7513**		−0.0282	−0.0037	**−0.7587**	0.6495		−0.0261	−0.0109	−0.9524	0.3015		0.0121	−0.0256	−0.9661	0.2541
(3s${}_{1/2}^{-1}$4d${}_{3/2}{)}_{1}$	0.0235	−0.0112	0.0287	0.0018		0.0233	−0.0274	−0.0114	−0.0021		0.0223	−0.0228	−0.0071	−0.0055		0.0221	−0.0217	−0.0063	−0.0065		0.0253	−0.0115	−0.0032	−0.0083		−0.0257	0.0086	−0.0028	−0.0085

**Table 6:** Types of sudden change of Shannon entropies, eigenlevel anticrossings (in a.u.), configuration mixing coefficients, and information exchanges for the levels in the subspace with $J^{P}=0^{e}$.

Z	Sudden Change	Eigenlevel Anticrossing	Configuration Mixing Coefficients
48	No	No	|2〉:−0.7060(3d${}_{5/2}^{-1}$4d${}_{5/2}{)}_{0}$+0.7071(3d${}_{3/2}^{-1}$4d${}_{3/2}{)}_{0}$, |3〉:−0.7030(3d${}_{5/2}^{-1}$4d${}_{5/2}{)}_{0}$−0.7022(3d${}_{3/2}^{-1}$4d${}_{3/2}{)}_{0}$
49	No	No	|2〉:−0.7102(3d${}_{5/2}^{-1}$4d${}_{5/2}{)}_{0}$+0.7029(3d${}_{3/2}^{-1}$4d${}_{3/2}{)}_{0}$, |3〉:−0.6987(3d${}_{5/2}^{-1}$4d${}_{5/2}{)}_{0}$−0.7063(3d${}_{3/2}^{-1}$4d${}_{3/2}{)}_{0}$

**Table 7:** Sudden change of Shannon entropies, eigenlevel anticrossings (in a.u.), configuration mixing coefficients, and information exchanges for the levels in the subspace with $J^{P}=1^{e}$.

Z	Sudden Change	Eigenlevel Anticrossing	Configuration Mixing Coefficients
37	No	$\Delta E_{11,10}=0.00615$	|10〉:0.9987(3p${}_{3/2}^{-1}$4f${}_{5/2}{)}_{1}$, |11〉:−0.9945(3s${}_{1/2}^{-1}$4s${}_{1/2}{)}_{1}$
38	Yes	No	|10〉:−0.9942(3s${}_{1/2}^{-1}$4s${}_{1/2}{)}_{1}$, |11〉:−0.9985(3p${}_{3/2}^{-1}$4f${}_{5/2}{)}_{1}$
			
41	No	No	|4〉:−0.6570(3d${}_{3/2}^{-1}$4d${}_{3/2}{)}_{1}$+0.6796(3d${}_{3/2}^{-1}$4d${}_{5/2}{)}_{1}$, |5〉:−0.7333(3d${}_{3/2}^{-1}$4d${}_{3/2}{)}_{1}$−0.6669(3d${}_{3/2}^{-1}$4d${}_{5/2}{)}_{1}$
42	No	No	|4〉:−0.6829(3d${}_{3/2}^{-1}$4d${}_{3/2}{)}_{1}$+0.6609(3d${}_{3/2}^{-1}$4d${}_{5/2}{)}_{1}$, |5〉:−0.7108(3d${}_{3/2}^{-1}$4d${}_{3/2}{)}_{1}$−0.6907(3d${}_{3/2}^{-1}$4d${}_{5/2}{)}_{1}$
43	No	No	|4〉:−0.7092(3d${}_{3/2}^{-1}$4d${}_{3/2}{)}_{1}$+0.6399(3d${}_{3/2}^{-1}$4d${}_{5/2}{)}_{1}$, |5〉:0.6862(3d${}_{3/2}^{-1}$4d${}_{3/2}{)}_{1}$+0.7151(3d${}_{3/2}^{-1}$4d${}_{5/2}{)}_{1}$
			
69	No	No	|10〉:−0.9827(3s${}_{1/2}^{-1}$4s${}_{1/2}{)}_{1}$, |11〉:−0.9989(3p${}_{3/2}^{-1}$4f${}_{5/2}{)}_{1}$
70	Yes	$\Delta E_{11,10}=0.0792$	|10〉:−0.9985(3p${}_{3/2}^{-1}$4f${}_{5/2}{)}_{1}$, |11〉:−0.9800(3s${}_{1/2}^{-1}$4s${}_{1/2}{)}_{1}$
			
74	No	No	|9〉:−0.9609(3p${}_{1/2}^{-1}$4p${}_{3/2}{)}_{1}$, |10〉:0.9971(3p${}_{3/2}^{-1}$4f${}_{5/2}{)}_{1}$
75	Yes	$\Delta E_{10,9}=0.0613$	|9〉:−0.7579(3p${}_{3/2}^{-1}$4f${}_{5/2}{)}_{1}$−0.6217(3p${}_{1/2}^{-1}$4p${}_{3/2}{)}_{1}$, |10〉:−0.6509(3p${}_{3/2}^{-1}$4f${}_{5/2}{)}_{1}$+0.7225(3p${}_{1/2}^{-1}$4p${}_{3/2}{)}_{1}$
			
80	Yes	$\Delta E_{11,10}=0.9544$	|10〉:−0.7513(3p${}_{1/2}^{-1}$4p${}_{3/2}{)}_{1}$−0.6581(3s${}_{1/2}^{-1}$4s${}_{1/2}{)}_{1}$, |11〉:−0.6593(3p${}_{1/2}^{-1}$4p${}_{3/2}{)}_{1}$+0.7513(3s${}_{1/2}^{-1}$4s${}_{1/2}{)}_{1}$
81	No	No	|10〉:−0.6494(3p${}_{1/2}^{-1}$4p${}_{3/2}{)}_{1}$−0.7587(3s${}_{1/2}^{-1}$4s${}_{1/2}{)}_{1}$, |11〉:−0.7599(3p${}_{1/2}^{-1}$4p${}_{3/2}{)}_{1}$+0.6495(3s${}_{1/2}^{-1}$4s${}_{1/2}{)}_{1}$
			
85	No	No	|8〉:0.3519(3p${}_{3/2}^{-1}$4f${}_{5/2}{)}_{1}$+0.9350(3p${}_{1/2}^{-1}$4p${}_{1/2}{)}_{1}$, |9〉:−0.9358(3p${}_{3/2}^{-1}$4f${}_{5/2}{)}_{1}$+0.3521(3p${}_{1/2}^{-1}$4p${}_{1/2}{)}_{1}$
86	Yes	$\Delta E_{9,8}=0.3714$	|8〉:−0.8901(3p${}_{3/2}^{-1}$4f${}_{5/2}{)}_{1}$−0.4545(3p${}_{1/2}^{-1}$4p${}_{1/2}{)}_{1}$, |9〉:−0.4552(3p${}_{3/2}^{-1}$4f${}_{5/2}{)}_{1}$+0.8897(3p${}_{1/2}^{-1}$4p${}_{1/2}{)}_{1}$
			
87	Yes	$\Delta E_{6,5}=0.2609$	|5〉:−0.8161(3d${}_{3/2}^{-1}$4d${}_{5/2}{)}_{1}$+0.5638(3p${}_{3/2}^{-1}$4p${}_{1/2}{)}_{1}$, |6〉:0.5734(3d${}_{3/2}^{-1}$4d${}_{5/2}{)}_{1}$+0.8156(3p${}_{3/2}^{-1}$4p${}_{1/2}{)}_{1}$
88	No	No	|5〉:−0.2570(3d${}_{3/2}^{-1}$4d${}_{5/2}{)}_{1}$+0.9534(3p${}_{3/2}^{-1}$4p${}_{1/2}{)}_{1}$, |6〉:−0.9638(3d${}_{3/2}^{-1}$4d${}_{5/2}{)}_{1}$−0.2633(3p${}_{3/2}^{-1}$4p${}_{1/2}{)}_{1}$
			
91	Yes	$\Delta E_{5,4}=0.3299$	|4〉:0.7755(3d${}_{3/2}^{-1}$4d${}_{3/2}{)}_{1}$+0.6212(3p${}_{3/2}^{-1}$4p${}_{1/2}{)}_{1}$, |5〉:−0.6294(3d${}_{3/2}^{-1}$4d${}_{3/2}{)}_{1}$+0.7726(3p${}_{3/2}^{-1}$4p${}_{1/2}{)}_{1}$
92	No	No	|4〉:0.3029(3d${}_{3/2}^{-1}$4d${}_{3/2}{)}_{1}$+0.9436(3p${}_{3/2}^{-1}$4p${}_{1/2}{)}_{1}$, |5〉:−0.9518(3d${}_{3/2}^{-1}$4d${}_{3/2}{)}_{1}$+0.3046(3p${}_{3/2}^{-1}$4p${}_{1/2}{)}_{1}$

**Table 8:** Sudden change of Shannon entropies, eigenlevel anticrossings (in a.u.), configuration mixing coefficients, and information exchanges for the levels in the mixed subspace with $J^{P}=2^{e}$ and 3*^e^*.

Z	Sudden Change	Eigenlevel Anticrossing	Configuration Mixing Coefficients
31	No	$\Delta E_{22,21}=3.0E-5$	|21〉:−0.9997(3p${}_{1/2}^{-1}$4f${}_{5/2}{)}_{2}$, |22〉:−0.9915(3p${}_{1/2}^{-1}$4f${}_{5/2}{)}_{3}$
32	Yes	No	|21〉:−0.9917(3p${}_{1/2}^{-1}$4f${}_{5/2}{)}_{3}$, |22〉:−0.9994(3p${}_{1/2}^{-1}$4f${}_{5/2}{)}_{2}$
			
33	No	No	|5〉:0.6393(3d${}_{5/2}^{-1}$4d${}_{3/2}{)}_{3}$−0.7477(3d${}_{5/2}^{-1}$4d${}_{5/2}{)}_{3}$, |6〉:−0.6056(3d${}_{5/2}^{-1}$4d${}_{3/2}{)}_{3}$−0.5745(3d${}_{5/2}^{-1}$4d${}_{5/2}{)}_{3}$
34	No	No	|5〉:0.6639(3d${}_{5/2}^{-1}$4d${}_{3/2}{)}_{3}$−0.7243(3d${}_{5/2}^{-1}$4d${}_{5/2}{)}_{3}$, |6〉:−0.5823(3d${}_{5/2}^{-1}$4d${}_{3/2}{)}_{3}$−0.6009(3d${}_{5/2}^{-1}$4d${}_{5/2}{)}_{3}$
35	No	No	|5〉:0.6879(3d${}_{5/2}^{-1}$4d${}_{3/2}{)}_{3}$−0.7009(3d${}_{5/2}^{-1}$4d${}_{5/2}{)}_{3}$, |6〉:−0.5671(3d${}_{5/2}^{-1}$4d${}_{3/2}{)}_{3}$−0.6296(3d${}_{5/2}^{-1}$4d${}_{5/2}{)}_{3}$
			|10〉:−0.9569(3d${}_{3/2}^{-1}$4d${}_{5/2}{)}_{3}$, |11〉:0.9791(3d${}_{3/2}^{-1}$4d${}_{3/2}{)}_{2}$
36	Yes	$\Delta E_{11,10}=0.00025$	|5〉:0.7118(3d${}_{5/2}^{-1}$4d${}_{3/2}{)}_{3}$−0.6768(3d${}_{5/2}^{-1}$4d${}_{5/2}{)}_{3}$, |6〉:−0.5557(3d${}_{5/2}^{-1}$4d${}_{3/2}{)}_{3}$−0.6598(3d${}_{5/2}^{-1}$4d${}_{5/2}{)}_{3}$
			|10〉:0.9791(3d${}_{3/2}^{-1}$4d${}_{3/2}{)}_{2}$, |11〉:−0.9590(3d${}_{3/2}^{-1}$4d${}_{5/2}{)}_{3}$
			|18〉:0.7865(3p${}_{3/2}^{-1}$4f${}_{5/2}{)}_{2}$+0.6159(3p${}_{3/2}^{-1}$4f${}_{7/2}{)}_{2}$, |19〉:0.9585(3p${}_{3/2}^{-1}$4f${}_{5/2}{)}_{3}$
37	Yes	$\Delta E_{19,18}=0.00049$	|18〉:0.9580(3p${}_{3/2}^{-1}$4f${}_{5/2}{)}_{3}$, |19〉:0.7641(3p${}_{3/2}^{-1}$4f${}_{5/2}{)}_{2}$+0.6434(3p${}_{3/2}^{-1}$4f${}_{7/2}{)}_{2}$
			
39	No	$\Delta E_{19,16}=1.62763$	|16〉:0.6809(3p${}_{3/2}^{-1}$4f${}_{5/2}{)}_{2}$−0.7281(3p${}_{3/2}^{-1}$4f${}_{7/2}{)}_{2}$, |19〉:−0.7294(3p${}_{3/2}^{-1}$4f${}_{5/2}{)}_{2}$−0.6824(3p${}_{3/2}^{-1}$4f${}_{7/2}{)}_{2}$
40	No	No	|16〉:0.6950(3p${}_{3/2}^{-1}$4f${}_{5/2}{)}_{2}$−0.7143(3p${}_{3/2}^{-1}$4f${}_{7/2}{)}_{2}$, |19〉:−0.7159(3p${}_{3/2}^{-1}$4f${}_{5/2}{)}_{2}$−0.6965(3p${}_{3/2}^{-1}$4f${}_{7/2}{)}_{2}$
41	No	No	|16〉:0.7069(3p${}_{3/2}^{-1}$4f${}_{5/2}{)}_{2}$−0.7022(3p${}_{3/2}^{-1}$4f${}_{7/2}{)}_{2}$, |19〉:−0.7042(3p${}_{3/2}^{-1}$4f${}_{5/2}{)}_{2}$−0.7083(3p${}_{3/2}^{-1}$4f${}_{7/2}{)}_{2}$
			
44	No	No	|9〉:−0.9716(3d${}_{3/2}^{-1}$4d${}_{5/2}{)}_{2}$, |10〉:0.9904(3d${}_{3/2}^{-1}$4d${}_{3/2}{)}_{2}$
45	Yes	$\Delta E_{10,9}=0.00075$	|9〉:−0.9772(3d${}_{3/2}^{-1}$4d${}_{3/2}{)}_{2}$, |10〉:−0.9647(3d${}_{3/2}^{-1}$4d${}_{5/2}{)}_{2}$
			
48	No	$\Delta E_{21,20}=0.0026$	|20〉:0.5823(3p${}_{1/2}^{-1}$4f${}_{5/2}{)}_{3}$−0.8073(3p${}_{1/2}^{-1}$4f${}_{7/2}{)}_{3}$, |21〉:−0.8116(3p${}_{1/2}^{-1}$4f${}_{5/2}{)}_{3}$−0.5808(3p${}_{1/2}^{-1}$4f${}_{7/2}{)}_{3}$
49	Yes	No	|20〉:0.7466(3p${}_{1/2}^{-1}$4f${}_{5/2}{)}_{3}$−0.6597(3p${}_{1/2}^{-1}$4f${}_{7/2}{)}_{3}$, |21〉:−0.6637(3p${}_{1/2}^{-1}$4f${}_{5/2}{)}_{3}$−0.7442(3p${}_{1/2}^{-1}$4f${}_{7/2}{)}_{3}$
			
63	No	No	|17〉:−0.6776(3p${}_{3/2}^{-1}$4f${}_{5/2}{)}_{3}$+0.7341(3p${}_{3/2}^{-1}$4f${}_{7/2}{)}_{3}$, |18〉:0.7349(3p${}_{3/2}^{-1}$4f${}_{5/2}{)}_{3}$+0.6775(3p${}_{3/2}^{-1}$4f${}_{7/2}{)}_{3}$
64	No	No	|17〉:−0.7226(3p${}_{3/2}^{-1}$4f${}_{5/2}{)}_{3}$+0.6900(3p${}_{3/2}^{-1}$4f${}_{7/2}{)}_{3}$, |18〉:0.6907(3p${}_{3/2}^{-1}$4f${}_{5/2}{)}_{3}$+0.7225(3p${}_{3/2}^{-1}$4f${}_{7/2}{)}_{3}$
			
72	No	No	|21〉:−0.9949(3p${}_{1/2}^{-1}$4f${}_{7/2}{)}_{3}$, |22〉:0.9925(3p${}_{1/2}^{-1}$4f${}_{5/2}{)}_{2}$
73	Yes	$\Delta E_{22,21}=0.0021$	|21〉:0.9924(3p${}_{1/2}^{-1}$4f${}_{5/2}{)}_{2}$, |22〉:0.9950(3p${}_{1/2}^{-1}$4f${}_{7/2}{)}_{3}$
			
75	Yes	$\Delta E_{16,15}=0.1307$	|15〉:0.5133(3p${}_{3/2}^{-1}$4f${}_{5/2}{)}_{2}$−0.7691(3p${}_{1/2}^{-1}$4p${}_{3/2}{)}_{2}$, |16〉:−0.7428(3p${}_{3/2}^{-1}$4f${}_{5/2}{)}_{2}$−0.6198(3p${}_{1/2}^{-1}$4p${}_{3/2}{)}_{2}$
		$\Delta E_{17,16}=0.1326$	|17〉:0.9710(3p${}_{3/2}^{-1}$4f${}_{5/2}{)}_{3}$, |18〉:0.9708(3p${}_{3/2}^{-1}$4f${}_{7/2}{)}_{3}$
76	Yes	$\Delta E_{18,17}=0.1121$	|15〉:−0.8822(3p${}_{3/2}^{-1}$4f${}_{5/2}{)}_{2}$+0.4528(3p${}_{3/2}^{-1}$4f${}_{7/2}{)}_{2}$, |16〉:0.9759(3p${}_{3/2}^{-1}$4f${}_{5/2}{)}_{3}$
		$\Delta E_{19,18}=0.2295$	|17〉:0.9757(3p${}_{3/2}^{-1}$4f${}_{7/2}{)}_{3}$, |18〉:0.3981(3p${}_{3/2}^{-1}$4f${}_{7/2}{)}_{2}$+0.8566(3p${}_{1/2}^{-1}$4p${}_{3/2}{)}_{2}$,|19〉:−0.7971(3p${}_{3/2}^{-1}$4f${}_{7/2}{)}_{2}$+0.4995(3p${}_{1/2}^{-1}$4p${}_{3/2}{)}_{2}$
77	No	No	|18〉:0.4395(3p${}_{3/2}^{-1}$4f${}_{5/2}{)}_{2}$+0.8704(3p${}_{3/2}^{-1}$4f${}_{7/2}{)}_{2}$, |19〉:0.9732(3p${}_{1/2}^{-1}$4p${}_{3/2}{)}_{2}$
			
86	Yes	No	|10〉:−0.9807(3d${}_{3/2}^{-1}$4d${}_{5/2}{)}_{2}$, |11〉:0.9991(3d${}_{3/2}^{-1}$4d${}_{5/2}{)}_{3}$,|12〉:−0.9768(3p${}_{3/2}^{-1}$4p${}_{1/2}{)}_{2}$
87	Yes	$\Delta E_{12,11}=0.0484$	|10〉:−0.4527(3d${}_{3/2}^{-1}$4d${}_{5/2}{)}_{2}$−0.8860(3p${}_{3/2}^{-1}$4p${}_{1/2}{)}_{2}$, |11〉:−0.8911(3d${}_{3/2}^{-1}$4d${}_{5/2}{)}_{2}$+0.4505(3p${}_{3/2}^{-1}$4p${}_{1/2}{)}_{2}$,|12〉:0.9992(3d${}_{3/2}^{-1}$4d${}_{5/2}{)}_{3}$
			
90	No	No	|9〉:−0.9260(3d${}_{3/2}^{-1}$4d${}_{3/2}{)}_{2}$−0.3756(3p${}_{3/2}^{-1}$4p${}_{1/2}{)}_{2}$, |10〉:0.3767(3d${}_{3/2}^{-1}$4d${}_{3/2}{)}_{2}$−0.9228(3p${}_{3/2}^{-1}$4p${}_{1/2}{)}_{2}$
91	Yes	$\Delta E_{9,8}=0.0073$	|8〉:0.9994(3d${}_{3/2}^{-1}$4d${}_{3/2}{)}_{3}$, |9〉:−0.4919(3d${}_{3/2}^{-1}$4d${}_{3/2}{)}_{2}$−0.8676(3p${}_{3/2}^{-1}$4p${}_{1/2}{)}_{2}$,|10〉:−0.8704(3d${}_{3/2}^{-1}$4d${}_{3/2}{)}_{2}$+0.4903(3p${}_{3/2}^{-1}$4p${}_{1/2}{)}_{2}$
92	No	No	|8〉:−0.9754(3p${}_{3/2}^{-1}$4p${}_{1/2}{)}_{2}$, |9〉:0.9994(3d${}_{3/2}^{-1}$4d${}_{3/2}{)}_{3}$

**Table 9:** Sudden change of Shannon entropies, eigenlevel anticrossings (in a.u.), configuration mixing coefficients, and information exchanges for the levels in the mixed subspace with $J^{P}=4^{e}$ and 5*^e^*.

Z	Sudden Change	Eigenlevel Anticrossing	Configuration Mixing Coefficients
35	No	No	|1〉:0.9999(3d${}_{5/2}^{-1}$4d${}_{5/2}{)}_{5}$, |2〉:0.9724(3d${}_{5/2}^{-1}$4d${}_{3/2}{)}_{4}$
36	Yes	$\Delta E_{2,1}=6.0E-5$	|1〉:0.9739(3d${}_{5/2}^{-1}$4d${}_{3/2}{)}_{4}$, |2〉:0.9999(3d${}_{5/2}^{-1}$4d${}_{5/2}{)}_{5}$
			
61	No	$\Delta E_{6,5}=3.0E-5$	|5〉:0.9998(3p${}_{3/2}^{-1}$4f${}_{7/2}{)}_{5}$, |6〉:0.9994(3p${}_{3/2}^{-1}$4f${}_{5/2}{)}_{4}$
62	Yes	No	|5〉:0.9994(3p${}_{3/2}^{-1}$4f${}_{5/2}{)}_{4}$, |6〉:0.9998(3p${}_{3/2}^{-1}$4f${}_{7/2}{)}_{5}$

**Table 10:** Sudden change of Shannon entropies, eigenlevel anticrossings (in a.u.), configuration mixing coefficients, and information exchanges for the levels in the subspace with $J^{P}=0^{o}$.

Z	Sudden Change	Eigenlevel Anticrossing	Configuration Mixing Coefficients
77	No	No	|3〉:0.5125(3p${}_{3/2}^{-1}$4d${}_{3/2}{)}_{0}$+0.8556(3p${}_{1/2}^{-1}$4s${}_{1/2}{)}_{0}$, |4〉:−0.8558(3p${}_{3/2}^{-1}$4d${}_{3/2}{)}_{0}$+0.5152(3p${}_{1/2}^{-1}$4s${}_{1/2}{)}_{0}$
78	Yes	$\Delta E_{4,3}=0.5284$	|3〉:−0.7783(3p${}_{3/2}^{-1}$4d${}_{3/2}{)}_{0}$−0.6230(3p${}_{1/2}^{-1}$4s${}_{1/2}{)}_{0}$, |4〉:−0.6242(3p${}_{3/2}^{-1}$4d${}_{3/2}{)}_{0}$+0.7807(3p${}_{1/2}^{-1}$4s${}_{1/2}{)}_{0}$

**Table 11:** Sudden change of Shannon entropies, eigenlevel anticrossings (in a.u.), configuration mixing coefficients, and information exchanges for the levels in the subspace with $J^{P}=1^{o}$.

Z	Sudden Change	Eigenlevel Anticrossing	Configuration Mixing Coefficients
41	No	No	|1〉:0.6517(3d${}_{5/2}^{-1}$4p${}_{3/2}{)}_{1}$−0.6373(3d${}_{3/2}^{-1}$4p${}_{1/2}{)}_{1}$, |2〉:0.6511(3d${}_{5/2}^{-1}$4p${}_{3/2}{)}_{1}$+0.7477(3d${}_{3/2}^{-1}$4p${}_{1/2}{)}_{1}$
42	No	No	|1〉:0.6399(3d${}_{5/2}^{-1}$4p${}_{3/2}{)}_{1}$−0.6645(3d${}_{3/2}^{-1}$4p${}_{1/2}{)}_{1}$, |2〉:0.6739(3d${}_{5/2}^{-1}$4p${}_{3/2}{)}_{1}$+0.7261(3d${}_{3/2}^{-1}$4p${}_{1/2}{)}_{1}$
43	No	No	|1〉:0.6246(3d${}_{5/2}^{-1}$4p${}_{3/2}{)}_{1}$−0.6928(3d${}_{3/2}^{-1}$4p${}_{1/2}{)}_{1}$, |2〉:0.6983(3d${}_{5/2}^{-1}$4p${}_{3/2}{)}_{1}$+0.7016(3d${}_{3/2}^{-1}$4p${}_{1/2}{)}_{1}$
44	No	No	|1〉:0.6057(3d${}_{5/2}^{-1}$4p${}_{3/2}{)}_{1}$−0.7216(3d${}_{3/2}^{-1}$4p${}_{1/2}{)}_{1}$, |2〉:0.7239(3d${}_{5/2}^{-1}$4p${}_{3/2}{)}_{1}$+0.6741(3d${}_{3/2}^{-1}$4p${}_{1/2}{)}_{1}$
			
49	Yes	$\Delta E_{7,6}=0.11137$	|6〉:−0.5121(3d${}_{5/2}^{-1}$4f${}_{7/2}{)}_{1}$−0.7024(3d${}_{3/2}^{-1}$4f${}_{5/2}{)}_{1}$+0.4840(3p${}_{3/2}^{-1}$4s${}_{1/2}{)}_{1}$, |7〉:0.2768(3d${}_{5/2}^{-1}$4f${}_{7/2}{)}_{1}$+0.3914(3d${}_{3/2}^{-1}$4f${}_{5/2}{)}_{1}$+0.8727(3p${}_{3/2}^{-1}$4s${}_{1/2}{)}_{1}$
50	Yes	No	|6〉:−0.2399(3d${}_{5/2}^{-1}$4f${}_{7/2}{)}_{1}$−0.3285(3d${}_{3/2}^{-1}$4f${}_{5/2}{)}_{1}$+0.9114(3p${}_{3/2}^{-1}$4s${}_{1/2}{)}_{1}$, |7〉:0.5245(3d${}_{5/2}^{-1}$4f${}_{7/2}{)}_{1}$+0.7383(3d${}_{3/2}^{-1}$4f${}_{5/2}{)}_{1}$+0.4063(3p${}_{3/2}^{-1}$4s${}_{1/2}{)}_{1}$
			
54	No	No	|5〉:0.7467(3d${}_{5/2}^{-1}$4f${}_{7/2}{)}_{1}$−0.5399(3d${}_{3/2}^{-1}$4f${}_{5/2}{)}_{1}$, |6〉:0.9920(3p${}_{3/2}^{-1}$4s${}_{1/2}{)}_{1}$
55	Yes	$\Delta E_{6,5}=0.01691$	|5〉:0.6020(3d${}_{5/2}^{-1}$4f${}_{7/2}{)}_{1}$−0.6605(3p${}_{3/2}^{-1}$4s${}_{1/2}{)}_{1}$, |6〉:0.4550(3d${}_{5/2}^{-1}$4f${}_{7/2}{)}_{1}$+0.7432(3p${}_{3/2}^{-1}$4s${}_{1/2}{)}_{1}$
56	No	No	|5〉:0.9906(3p${}_{3/2}^{-1}$4s${}_{1/2}{)}_{1}$, |6〉:0.7531(3d${}_{5/2}^{-1}$4f${}_{7/2}{)}_{1}$−0.5327(3d${}_{3/2}^{-1}$4f${}_{5/2}{)}_{1}$
			
58	No	No	|4〉:0.9012(3d${}_{5/2}^{-1}$4f${}_{5/2}{)}_{1}$−0.3334(3d${}_{5/2}^{-1}$4f${}_{7/2}{)}_{1}$, |5〉:0.9662(3p${}_{3/2}^{-1}$4s${}_{1/2}{)}_{1}$
59	Yes	$\Delta E_{5,4}=0.05159$	|4〉:−0.4396(3d${}_{5/2}^{-1}$4f${}_{5/2}{)}_{1}$+0.8847(3p${}_{3/2}^{-1}$4s${}_{1/2}{)}_{1}$, |5〉:0.8176(3d${}_{5/2}^{-1}$4f${}_{5/2}{)}_{1}$+0.4599(3p${}_{3/2}^{-1}$4s${}_{1/2}{)}_{1}$
			
70	No	No	|11〉:0.7800(3p${}_{1/2}^{-1}$4d${}_{3/2}{)}_{1}$+0.6237(3s${}_{1/2}^{-1}$4p${}_{1/2}{)}_{1}$, |12〉:0.6248(3p${}_{1/2}^{-1}$4d${}_{3/2}{)}_{1}$−0.7794(3s${}_{1/2}^{-1}$4p${}_{1/2}{)}_{1}$
71	Yes	$\Delta E_{12,11}=0.6555$	|11〉:0.7017(3p${}_{1/2}^{-1}$4d${}_{3/2}{)}_{1}$+0.7105(3s${}_{1/2}^{-1}$4p${}_{1/2}{)}_{1}$, |12〉:0.7116(3p${}_{1/2}^{-1}$4d${}_{3/2}{)}_{1}$−0.7013(3s${}_{1/2}^{-1}$4p${}_{1/2}{)}_{1}$
			
78	Yes	$\Delta E_{9,8}=0.3099$	|8〉:0.6951(3p${}_{3/2}^{-1}$4d${}_{3/2}{)}_{1}$−0.6995(3p${}_{1/2}^{-1}$4s${}_{1/2}{)}_{1}$, |9〉:−0.7083(3p${}_{3/2}^{-1}$4d${}_{3/2}{)}_{1}$−0.7049(3p${}_{1/2}^{-1}$4s${}_{1/2}{)}_{1}$
79	No	No	|8〉:0.9353(3p${}_{3/2}^{-1}$4d${}_{3/2}{)}_{1}$−0.3173(3p${}_{1/2}^{-1}$4s${}_{1/2}{)}_{1}$, |9〉:0.3352(3p${}_{3/2}^{-1}$4d${}_{3/2}{)}_{1}$+0.9346(3p${}_{1/2}^{-1}$4s${}_{1/2}{)}_{1}$
			
81	Yes	$\Delta E_{10,9}=0.2760$	|9〉:0.6276(3p${}_{3/2}^{-1}$4d${}_{5/2}{)}_{1}$+0.7589(3p${}_{1/2}^{-1}$4s${}_{1/2}{)}_{1}$, |10〉:0.7684(3p${}_{3/2}^{-1}$4d${}_{5/2}{)}_{1}$−0.6393(3p${}_{1/2}^{-1}$4s${}_{1/2}{)}_{1}$
82	No	No	|9〉:0.9396(3p${}_{3/2}^{-1}$4d${}_{5/2}{)}_{1}$+0.3119(3p${}_{1/2}^{-1}$4s${}_{1/2}{)}_{1}$, |10〉:−0.3212(3p${}_{3/2}^{-1}$4d${}_{5/2}{)}_{1}$+0.9453(3p${}_{1/2}^{-1}$4s${}_{1/2}{)}_{1}$

**Table 12:** Sudden change of Shannon entropies, eigenlevel anticrossings (in a.u.), configuration mixing coefficients, and information exchanges for the levels in the subspace with $J^{P}=2^{o}$.

Z	Sudden Change	Eigenlevel Anticrossing	Configuration Mixing Coefficients
44	No	No	|10〉:0.6811(3p${}_{3/2}^{-1}$4d${}_{3/2}{)}_{2}$−0.7233(3p${}_{3/2}^{-1}$4d${}_{5/2}{)}_{2}$, |11〉:−0.7291(3p${}_{3/2}^{-1}$4d${}_{3/2}{)}_{2}$−0.6813(3p${}_{3/2}^{-1}$4d${}_{5/2}{)}_{2}$
45	No	No	|10〉:0.7152(3p${}_{3/2}^{-1}$4d${}_{3/2}{)}_{2}$−0.6903(3p${}_{3/2}^{-1}$4d${}_{5/2}{)}_{2}$, |11〉:−0.6958(3p${}_{3/2}^{-1}$4d${}_{3/2}{)}_{2}$−0.7151(3p${}_{3/2}^{-1}$4d${}_{5/2}{)}_{2}$
			
52	Yes	$\Delta E_{9,8}=0.02944$	|7〉:0.3340(3d${}_{3/2}^{-1}$4f${}_{5/2}{)}_{2}$−0.8993(3d${}_{3/2}^{-1}$4f${}_{7/2}{)}_{2}$, |8〉:0.9396(3d${}_{3/2}^{-1}$4f${}_{5/2}{)}_{2}$+0.3376(3d${}_{3/2}^{-1}$4f${}_{7/2}{)}_{2}$, |9〉:−0.2008(3d${}_{3/2}^{-1}$4f${}_{7/2}{)}_{2}$−0.9773(3p${}_{3/2}^{-1}$4s${}_{1/2}{)}_{2}$
53	Yes	$\Delta E_{8,7}=0.05352$	|7〉:0.4179(3d${}_{3/2}^{-1}$4f${}_{7/2}{)}_{2}$−0.8822(3p${}_{3/2}^{-1}$4s${}_{1/2}{)}_{2}$, |8〉:−0.8135(3d${}_{3/2}^{-1}$4f${}_{7/2}{)}_{2}$−0.4619(3p${}_{3/2}^{-1}$4s${}_{1/2}{)}_{2}$, |9〉:0.9320(3d${}_{3/2}^{-1}$4f${}_{5/2}{)}_{2}$+0.3604(3d${}_{3/2}^{-1}$4f${}_{7/2}{)}_{2}$
			
56	No	No	|5〉:0.5801(3d${}_{5/2}^{-1}$4f${}_{5/2}{)}_{2}$−0.7726(3d${}_{5/2}^{-1}$4f${}_{7/2}{)}_{2}$, |6〉:−0.8089(3d${}_{5/2}^{-1}$4f${}_{5/2}{)}_{2}$−0.5804(3d${}_{5/2}^{-1}$4f${}_{7/2}{)}_{2}$, |7〉:−0.2122(3d${}_{5/2}^{-1}$4f${}_{7/2}{)}_{2}$−0.9739(3p${}_{3/2}^{-1}$4s${}_{1/2}{)}_{2}$
57	Yes	$\Delta E_{6,5}=0.0630$	|5〉:0.5041(3d${}_{5/2}^{-1}$4f${}_{5/2}{)}_{2}$−0.6690(3d${}_{5/2}^{-1}$4f${}_{7/2}{)}_{2}$+0.5234(3p${}_{3/2}^{-1}$4s${}_{1/2}{)}_{2}$, |6〉:0.3939(3d${}_{5/2}^{-1}$4f${}_{5/2}{)}_{2}$−0.3566(3d${}_{5/2}^{-1}$4f${}_{7/2}{)}_{2}$−0.8445(3p${}_{3/2}^{-1}$4s${}_{1/2}{)}_{2}$, |7〉:0.7646(3d${}_{5/2}^{-1}$4f${}_{5/2}{)}_{2}$+0.6371(3d${}_{5/2}^{-1}$4f${}_{7/2}{)}_{2}$
		$\Delta E_{7,6}=0.0486$	
58	Yes	No	|5〉:0.2407(3d${}_{5/2}^{-1}$4f${}_{7/2}{)}_{2}$−0.9516(3p${}_{3/2}^{-1}$4s${}_{1/2}{)}_{2}$, |6〉:0.6200(3d${}_{5/2}^{-1}$4f${}_{5/2}{)}_{2}$−0.7145(3d${}_{5/2}^{-1}$4f${}_{7/2}{)}_{2}$, |7〉:0.7635(3d${}_{5/2}^{-1}$4f${}_{5/2}{)}_{2}$+0.6432(3d${}_{5/2}^{-1}$4f${}_{7/2}{)}_{2}$
			
61	No	No	|6〉:0.6937(3d${}_{5/2}^{-1}$4f${}_{5/2}{)}_{2}$−0.7067(3d${}_{5/2}^{-1}$4f${}_{7/2}{)}_{2}$, |7〉:0.7165(3d${}_{5/2}^{-1}$4f${}_{5/2}{)}_{2}$+0.6957(3d${}_{5/2}^{-1}$4f${}_{7/2}{)}_{2}$
62	No	No	|6〉:0.7143(3d${}_{5/2}^{-1}$4f${}_{5/2}{)}_{2}$−0.6881(3d${}_{5/2}^{-1}$4f${}_{7/2}{)}_{2}$, |7〉:0.6964(3d${}_{5/2}^{-1}$4f${}_{5/2}{)}_{2}$+0.7157(3d${}_{5/2}^{-1}$4f${}_{7/2}{)}_{2}$
			
69	No	No	|8〉:−0.6806(3d${}_{3/2}^{-1}$4f${}_{5/2}{)}_{2}$+0.7281(3d${}_{3/2}^{-1}$4f${}_{7/2}{)}_{2}$, |9〉:−0.7318(3d${}_{3/2}^{-1}$4f${}_{5/2}{)}_{2}$−0.6801(3d${}_{3/2}^{-1}$4f${}_{7/2}{)}_{2}$
70	No	No	|8〉:−0.7142(3d${}_{3/2}^{-1}$4f${}_{5/2}{)}_{2}$+0.6957(3d${}_{3/2}^{-1}$4f${}_{7/2}{)}_{2}$, |9〉:−0.6991(3d${}_{3/2}^{-1}$4f${}_{5/2}{)}_{2}$−0.7136(3d${}_{3/2}^{-1}$4f${}_{7/2}{)}_{2}$

**Table 13:** Sudden change of Shannon entropies, eigenlevel anticrossings (in a.u.), configuration mixing coefficients, and information exchanges for the levels in the mixed subspace with $J^{P}=3^{o}$ and $4^{o}$.

Z	Sudden Change	Eigenlevel Anticrossing	Configuration Mixing Coefficients
35	No	No	|13〉:0.9995(3p${}_{3/2}^{-1}$4d${}_{5/2}{)}_{4}$, |14〉:0.9917(3p${}_{3/2}^{-1}$4d${}_{3/2}{)}_{3}$
36	Yes	$\Delta E_{14,13}=0.00023$	|13〉:0.9918(3p${}_{3/2}^{-1}$4d${}_{3/2}{)}_{3}$, |14〉:0.9995(3p${}_{3/2}^{-1}$4d${}_{5/2}{)}_{4}$
			
39	No	$\Delta E_{18,17}=4.0E-5$	|17〉:−0.9997(3s${}_{1/2}^{-1}$4f${}_{7/2}{)}_{4}$, |18〉:−0.7589(3s${}_{1/2}^{-1}$4f${}_{5/2}{)}_{3}$+0.6508(3s${}_{1/2}^{-1}$4f${}_{7/2}{)}_{3}$
40	Yes	No	|17〉:−0.7618(3s${}_{1/2}^{-1}$4f${}_{5/2}{)}_{3}$+0.6473(3s${}_{1/2}^{-1}$4f${}_{7/2}{)}_{3}$, |18〉:−0.9997(3s${}_{1/2}^{-1}$4f${}_{7/2}{)}_{4}$
			
41	No	No	|5〉:−0.6937(3d${}_{5/2}^{-1}$4f${}_{5/2}{)}_{3}$+0.7150(3d${}_{5/2}^{-1}$4f${}_{7/2}{)}_{3}$, |8〉:0.7158(3d${}_{5/2}^{-1}$4f${}_{5/2}{)}_{3}$+0.6776(3d${}_{5/2}^{-1}$4f${}_{7/2}{)}_{3}$
42	No	No	|5〉:−0.7048(3d${}_{5/2}^{-1}$4f${}_{5/2}{)}_{3}$+0.7045(3d${}_{5/2}^{-1}$4f${}_{7/2}{)}_{3}$, |8〉:0.7048(3d${}_{5/2}^{-1}$4f${}_{5/2}{)}_{3}$+0.6891(3d${}_{5/2}^{-1}$4f${}_{7/2}{)}_{3}$
43	No	No	|5〉:−0.7156(3d${}_{5/2}^{-1}$4f${}_{5/2}{)}_{3}$+0.6941(3d${}_{5/2}^{-1}$4f${}_{7/2}{)}_{3}$, |8〉:0.6940(3d${}_{5/2}^{-1}$4f${}_{5/2}{)}_{3}$+0.7005(3d${}_{5/2}^{-1}$4f${}_{7/2}{)}_{3}$
			
52	No	No	|6〉:−0.6863(3d${}_{5/2}^{-1}$4f${}_{5/2}{)}_{4}$+0.7233(3d${}_{5/2}^{-1}$4f${}_{7/2}{)}_{4}$, |7〉:−0.7156(3d${}_{5/2}^{-1}$4f${}_{5/2}{)}_{4}$−0.6860(3d${}_{5/2}^{-1}$4f${}_{7/2}{)}_{4}$
53	No	No	|6〉:−0.7282(3d${}_{5/2}^{-1}$4f${}_{5/2}{)}_{4}$+0.6811(3d${}_{5/2}^{-1}$4f${}_{7/2}{)}_{4}$, |7〉:−0.6744(3d${}_{5/2}^{-1}$4f${}_{5/2}{)}_{4}$−0.7283(3d${}_{5/2}^{-1}$4f${}_{7/2}{)}_{4}$
			
56	No	$\Delta E_{12,11}=2.0E-5$	|11〉:−0.9836(3d${}_{3/2}^{-1}$4f${}_{7/2}{)}_{4}$, |12〉:−0.9577(3d${}_{3/2}^{-1}$4f${}_{5/2}{)}_{3}$
57	Yes	No	|11〉:−0.9497(3d${}_{3/2}^{-1}$4f${}_{5/2}{)}_{3}$, |12〉:−0.9843(3d${}_{3/2}^{-1}$4f${}_{7/2}{)}_{4}$
			
63	No	$\Delta E_{11,10}=0.0274$	|10〉:0.6411(3d${}_{3/2}^{-1}$4f${}_{5/2}{)}_{3}$+0.7667(3d${}_{3/2}^{-1}$4f${}_{7/2}{)}_{3}$, |11〉:−0.7662(3d${}_{3/2}^{-1}$4f${}_{5/2}{)}_{3}$+0.6385(3d${}_{3/2}^{-1}$4f${}_{7/2}{)}_{3}$
64	Yes	No	|10〉:0.7250(3d${}_{3/2}^{-1}$4f${}_{5/2}{)}_{3}$+0.6882(3d${}_{3/2}^{-1}$4f${}_{7/2}{)}_{3}$, |11〉:−0.6875(3d${}_{3/2}^{-1}$4f${}_{5/2}{)}_{3}$+0.7227(3d${}_{3/2}^{-1}$4f${}_{7/2}{)}_{3}$

**Table 14:** Sudden change of Shannon entropies, eigenlevel anticrossings (in a.u.), configuration mixing coefficients, and information exchanges for the levels in the mixed subspace with $J^{P}=5^{o}$ and $6^{o}$.

Z	Sudden Change	Eigenlevel Anticrossing	Configuration Mixing Coefficients
56	Yes	$\Delta E_{2,1}=0.00028$	|1〉:(3d${}_{5/2}^{-1}$4f${}_{7/2}{)}_{6}$, |2〉:0.9922(3d${}_{5/2}^{-1}$4f${}_{5/2}{)}_{5}$
57	No	No	|1〉:0.9926(3d${}_{5/2}^{-1}$4f${}_{5/2}{)}_{5}$, |2〉:(3d${}_{5/2}^{-1}$4f${}_{7/2}{)}_{6}$
